# Advanced analysis of disintegrating pharmaceutical compacts using deep learning-based segmentation of time-resolved micro-tomography images

**DOI:** 10.1016/j.heliyon.2024.e26025

**Published:** 2024-02-12

**Authors:** Samuel Waldner, Erwin Wendelspiess, Pascal Detampel, Christian M. Schlepütz, Jörg Huwyler, Maxim Puchkov

**Affiliations:** a Department of Pharmaceutical Sciences, Division of Pharmaceutical Technology, University of Basel, Klingelberstrasse 50, 4056, Basel, Switzerland; b Swiss Light Source, Paul Scherrer Institute, 5232, Villigen PSI, Switzerland

**Keywords:** Disintegration, Swelling, Tablets, Time-resolved micro-computed tomography, Deep learning-based image segmentation

## Abstract

The mechanism governing pharmaceutical tablet disintegration is far from fully understood. Despite the importance of controlling a formulation's disintegration process to maximize the active pharmaceutical ingredient's bioavailability and ensure predictable and consistent release profiles, the current understanding of the process is based on indirect or superficial measurements. Formulation science could, therefore, additionally deepen the understanding of the fundamental physical principles governing disintegration based on direct observations of the process. We aim to help bridge the gap by generating a series of time-resolved X-ray micro-computed tomography (μCT) images capturing volumetric images of a broad range of mini-tablet formulations undergoing disintegration. Automated image segmentation was a prerequisite to overcoming the challenges of analyzing multiple time series of heterogeneous tomographic images at high magnification. We devised and trained a convolutional neural network (CNN) based on the U-Net architecture for autonomous, rapid, and consistent image segmentation. We created our own μCT data reconstruction pipeline and parameterized it to deliver image quality optimal for our CNN-based segmentation. Our approach enabled us to visualize the internal microstructures of the tablets during disintegration and to extract parameters of disintegration kinetics from the time-resolved data. We determine by factor analysis the influence of the different formulation components on the disintegration process in terms of both qualitative and quantitative experimental responses. We relate our findings to known formulation component properties and established experimental results. Our direct imaging approach, enabled by deep learning-based image processing, delivers new insights into the disintegration mechanism of pharmaceutical tablets.

## Graphical abstract

**Figure:**
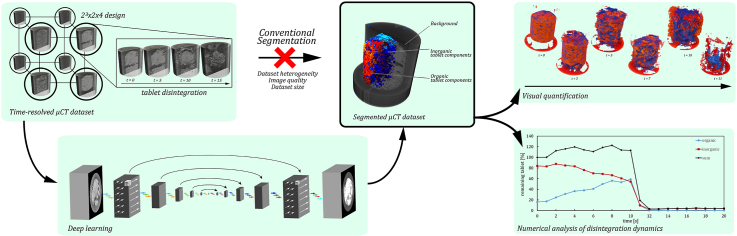

## 1 Introduction

Pharmaceutical tablet disintegration, the process by which a tablet breaks apart into fragments in the desired manner upon initial contact with gastrointestinal fluids, is crucial for the bioavailability of drugs and ensuring consistent release behavior of the active pharmaceutical ingredient (API) for modified release formulations. For fast-releasing tablets, the desired disintegration profiles are achieved by ensuring rapid fragmentation of the compact to increase the surface exposed to the dissolution medium. The rate and extent of fragmentation need to be precisely limited for slow-release formulations. Despite its importance, understanding of the disintegration mechanism remains incomplete [1].

Many approaches have been used to characterize the processes proposed to play a role in overcoming the inter-particle forces that hold the compact together and thus lead to disintegration [1,2]. In the common understanding, disintegration starts with and is sustained by liquid penetration into the porous domain of the tablet [3,4]. Therefore, the compact's porosity plays an important role, as well as the presence of a percolating network of material that conducts water throughout the tablet for rapid disintegration. One such water transportation process is called wicking [5]. Wicking is followed by swelling, a commonly accepted aspect of the disintegration mechanism whereby a disintegrant polymer omnidirectionally expands, with water acting as a plasticizer [6,7]. Additionally, unidirectional elastic recovery of deformation-induced strains upon contact with water has been put forward as a mechanism of action for certain disintegrants [8,9]. Other, more contended disintegration mechanisms have been proposed, for example, pressure buildup in the porous domain resulting from entrapped air heating up due to wetting of the pores [1,10]. Another controversial disintegration mechanism hypothesized for starches is particle-particle repulsion resulting from water attenuating hydrogen bonds, van der Waals and electrostatic forces, and subsequent induction of a repulsive force between the particles [11]. The complexity of the disintegration mechanism and difficulty quantifying it arise from the complex interplay of different formulation components with various functions based on their physicochemical properties. These properties must be investigated in isolation, combined with the other formulation components, and with the disintegration medium to quantify their effect.

So far, the quantification of disintegration was either based on superficial observation, static measurements, or indirect detection of the processes occurring within the tablet. Experiments include, for example, simultaneous water uptake rate and disintegration force measurements [12]; direct pore network imaging using micro-computed tomography (μCT) [13,14]; an indirect measure of bulk compact porosity using terahertz time-domain spectroscopy [15,16]; application of high-speed video recording to visualize disintegrant in action [8]; application of high-speed MRI imaging to quantify water distribution and swelling during the disintegration process [[17], [18], [19], [20], [21]]; methods to predict disintegration behavior based on density distribution measurements using ultrasound amongst other compact properties [22]; water-front and internal defect measurements using terahertz pulsed imaging [23,24]; characterization of compact fragments resulting from disintegration using focused beam reflectance measurements [25,26]; dynamic laser diffraction [[27], [28], [29]]; or video recordings [8,30]. Existing research thus covers many aspects of this complex process. Due to the process's complexity, interpreting indirectly obtained or isolated results can often lead to misinterpretation of the underlying mechanism. Time-resolved X-ray micro-computed tomography is an imaging technique that acquires direct visual data at high spatial and temporal resolutions, making it possible to observe and quantify the complete process.

Static X-ray μCT [[31], [32], [33]] is a powerful, non-destructive, high-resolution volumetric imaging technique that provides valuable insight into the internal microstructure of visually opaque samples. Thus, μCT is applied across a broad range of research fields, from earth science [34], medical research [35], and material science [36] to pharmaceutical research [37,38]. The use of synchrotron radiation [32] enables phase-contrast imaging [39]. Resolutions in the submicron [40] down to the nanometer (i.e., less than 100 nm) ranges [41] become attainable thanks to the parallel, coherent, and high-brilliance X-rays produced by synchrotron facilities. The high flux of these X-ray sources also provides the possibility of acquiring time-resolved image sequences of dynamic processes through the rapid and consecutive acquisition of CT scans, i.e., time-resolved μCT.

Time-resolved μCT has been applied to study foam systems [42,43], fluid interactions with porous geological samples [44,45], or to observe the mechanics involved in a wingbeat of the blowfly [46]. Further extending visual analysis with computer-aided techniques requires consistent segmentation of the dynamic volumetric data, which needs consistent imaging quality throughout the entire acquisition. Enhancing the image quality of μCT imaging in second- or sub-second time regimes [47] is possible with techniques like phase-contrast enhancement [48]. Such strategies are essential at high acquisition rates to reduce the dose imparted by the X-rays to levels that will not damage the sample [49]. Sometimes, polychromatic X-ray radiation is preferable over monochromic to reach the flux intensity necessary for adequate image quality despite the beam hardening artifacts [34].

The appearance of motion artifacts is a known problem for time-resolved μCT. Any sample motion during a given CT acquisition step violates the base assumption of reconstruction algorithms that the sample remains static [50], thus introducing motion artifacts during reconstruction of the three-dimensional (3D) image out of the projection raw data. Simply increasing the acquisition rate is rarely viable due to the limitations coming from insufficient beam intensity [49]. Higher acquisition frequencies could also introduce distortions in the studied process as rotation-related forces constantly act on the sample during image acquisition [34]. The data volumes produced are another challenge, as higher acquisition rates generate several gigabytes per second of data streams. Therefore, specialized hardware and software are required for data acquisition, transfer, storage, image reconstruction, and analysis [51].

Image analysis, in general, plays an important role in various pharmaceutical applications, most prominently in quality control for manufacturing pharmaceutical dosage forms [52]. Examples include optical imaging as a non-destructive alternative to established techniques for predicting tablet hardness and other quality attributes of pharmaceutical compacts [53]. Optical imaging has also been applied to rapidly assess coating thickness on pellets by quantifying the color information of a dye in the coating layer [54]. X-ray-based imaging can be used as a non-destructive way of semi-quantitatively determining drug content by measuring the distribution and content of pellets within multiparticulate tablets [55] or by studying the drug particle distribution within tablets [37]. However, fully automated image analysis of complex objects, such as pharmaceutical tablet formulations during disintegration, remains an unsolved challenge for which approaches based on machine learning have been a promising development.

Machine learning applications in image analysis are slowly entering pharmaceutical research [[56], [57], [58]]. Deep learning [59], in particular, is a tool that has been successfully employed in computer vision tasks to overcome the severe limitations of standard image analysis, especially in terms of object recognition, image data classification, and feature extraction from complex and heterogeneous datasets. Medical imaging is a field where the application of deep learning shows promising results. Some recent applications include the classification of optical images as an automated skin cancer diagnostic tool [60], in radiology for automated breast cancer screening [61], and in predicting the progression of the severity of COVID-19 in patients based on computed tomography scans [62]. In all these examples, researchers reported that the diagnostic performance of the deep-learning algorithms was similar to or better than that of trained professionals.

Convolutional Neural Networks (CNNs) [63,64] are a type of deep learning algorithm specialized in image processing using multiple consecutive layers of convolution operations for visual feature extraction. Image classification networks, like the GoogleNet Inception v3 CNN architecture [65], use these features for classification by coupling the output of the convolutional layers to a fully connected layer with an activation function. Similarly, image segmentation network architectures like the U-Net [66] connect the result of the convolution layers to a set of consecutive deconvolution layers. These then use the extracted feature information to build up a segmented version of the input image. Convolution operations make CNNs uniquely suited for and thus widely applied in image analysis, as the convolutions inherently process spatial information in addition to color information. CNNs can be adapted to use 3D convolution operations, which allows for the processing of 3D images from computed tomography scans [67], making CNNs a natural choice for the post-processing of μCT data.

Thus far, CNNs have been successfully applied to analyze static μCT data. Examples include the automated high-throughput extraction of morphological traits from μCT images of rice plants [68], geological sample classification [69], the classification of urinary stones from medical μCT scans [70], permeability estimation of complex pore structures of carbonate rock [71], and the quality enhancement of low-dose μCT scans [72]. In pharmaceutical research, CNNs have been applied to predict mini-tablet dissolution performance based on film coating integrity analysis using convolutional neural networks [73].

In this work, we extend the application of CNNs to the post-processing of dynamic μCT data. We prepared a comprehensive set of pharmaceutical tablets and acquired time-resolved μCT data that captured the disintegration of each formulation individually. CNN-based image segmentation allowed us to apply computer-aided and human expert-assisted analysis to the data and to illustrate our findings with videos showing the internal structures of each tablet during disintegration. We use factor and response surface analysis to assess the influence of formulation component properties on disintegration, and we relate the results of our direct measurements of tablets to established research. We provide our processed time-resolved μCT dataset for further research and our full software pipeline to make it easy to apply this imaging technique to other areas of pharmaceutical research.

## 2 Materials and methods

### 2.1 Composition of mini-tablets

A set of 64 mini-tablets with compositions according to a full-factorial design of experiments (DoE) generated in the STAVEX software (Aicos Technologies, Switzerland) was produced (see supporting information Table S1 and Table S2). The design of the experiments was a blocked qualitative full-factorial design (2^3^ × 2 × 4). Each formulation consisted of an API, a disintegration-modifying polymer (either a superdisintegrant or a swelling polymer), a lubricant, and a filler material.

The following compounds were chosen for their widespread use and their representative physicochemical properties: Caffeine (HPLC grade, Sigma-Aldrich, Switzerland), oxantel pamoate (Megafine Pharma Ltd., India), croscarmellose sodium (Ac-Di-Sol SD-711, FMC Corporation, Pennsylvania, USA), sodium starch glycolate (EXPLOTAB, JRS Pharma, Germany), hydroxypropyl methylcellulose (Methocel E4, Prochem, Switzerland), polyethylene oxide (WSR 301, Colorcon, Pennsylvania, USA), magnesium stearate (Hänseler AG, Switzerland), sodium stearyl fumarate (LubriSanaq, Pharmatrans Sanaq, Switzerland), (Fuji Chemical Industries Co., Ltd., Japan), Mannitol (Parteck M 300, Merck, Germany), microcrystalline cellulose (Avicel 102, FMC BioPolymer, Germany) and functionalized calcium carbonate (OMYAPHARM OG, OMYA AG, Switzerland).

The drug load was set to 10% (w/w) for all formulations. The super-disintegrants croscarmellose sodium and sodium starch glycolate were included at 3% (w/w) concentration, whereas the gel-forming polymers hydroxypropyl methylcellulose and polyethylene oxide contents were set to 40% (w/w). The filler material comprised 85% (w/w) of rapid-release formulations and 48% (w/w) of swelling formulations. All formulations contained 2% (w/w) of lubricant.

Apparent true densities of all formulation components were measured using an AccuPy 1330 helium pycnometer (One Micromeritics, Georgia, USA).

### 2.2 Production of mini-tablets

Before compaction, powder blends were mixed in a tumble-action mixer (Turbula type T2C, Willy A. Bachofen, Switzerland) for 10 min.

Mini-tablets were compacted using a 2 mm multi-tip tooling with a 1.4 mm curvature radius on a Styl’One Classic compaction simulator (Medel’Pharm, France). Compression time was set to 300 ms, and relaxation time to 39 ms. A fill height of 8 mm was used, and the maximum compression force was set to 5.5 kN. The die was filled manually before each compaction. Upper and lower punch forces were recorded (Table S3).

The mini-tablets were characterized in thickness, weight, and porosity (Table S5).

### 2.3 Experimental setup of time-resolved μCT acquisition

Four-dimensional phase-contrast μCT images were acquired at the TOMCAT beamline X02DA of the Swiss Light Source synchrotron facility at the Paul Scherrer Institute (Villigen, Switzerland). Due to the high X-ray density of the samples, polychromatic X-rays with a peak energy of around 26 keV were used for imaging. A 5 mm glassy carbon power filter plus 1.525 mm of Si were used to pre-harden the 2.9 T bending magnet beam and avoid excessive radiation damage to the sample and optics. The propagation distance from the sample to the detector was 0.6 m. The attenuated X-rays were converted to visible light using a 100 μm thick LuAG:Ce scintillator. The resulting X-ray projection images were captured using the custom-built GigaFRoST [51] detector and readout system coupled to a white-beam compatible microscope (Optique Peter, Lentilly, France) at a magnification of 6.8×. The effective pixel size of this setup was 1.612 μm^2^.

The mini-tablets were mounted on the sample stage using a custom 3D-printed tablet holder. The holder was printed with Form-2 stereolithographic printer (Formlabs, Massachusetts, USA, using Formlabs' ‘clear’ polymer) and was designed to keep the tablet in place during the disintegration process with minimal X-ray beam obscuration. Degassed ultrapure water (18.2 MΩ cm) was introduced directly above the tablets using a syringe pump (Microfluidic dual programmable Pico 11 Plus Elite, Harvard Apparatus, United States) at 6 mLs^−1^. The pump was triggered automatically upon image acquisition start after one complete dry scan of each tablet. Images of the experimental setup and schematics of the tablet holder can be found in the supporting information, Fig. S1.

Each tablet was imaged once before water was introduced. The initial disintegration phase was imaged at an acquisition rate of 1 scan per second. After a set time period of either 10 or 30 s, depending on the formulation, the sampling rate was lowered to 1 scan per 5 s, down to a minimum of 1 scan per 120 s. Image acquisition was stopped early if no part of the tablet remained in the camera's field of view before the total scan time elapsed. For some tablets that disintegrated very fast (i.e., in less than 10 s), scans were acquired with a smaller field of view but at higher rates, up to 2 Hz. For details on scan parameters, including acquisition rates, see supporting information in Table S6.

Each acquisition consisted of 500 projections with 1 ms exposure time at angles evenly spaced over 180° with a projection size of 2016 × 1800 px (pixels), resulting in a reconstructed stack dimension of 2016 × 2016 × 1800 voxels. Each run was preceded by acquiring 50 dark and 100 flat images for projection normalization.

### 2.4 Image reconstruction

Our image reconstruction and segmentation software pipeline operations are separated based on differing hardware requirements. Each step could be initiated on the computation cluster with optimized hardware parameters.

The software pipeline [74] is divided into the following steps:

1. Flat-field correction of the raw projection data using flat field and dark field images utilizing the tomopy library [75].
2. Phase-contrast information extraction using a Paganin filter [39] and computation of the negative log of the image array, implemented in CUDA C++ using the ArrayFire library [76] to run on graphics processing units (GPUs)
3. Stripe removal using a Fourier-Wavelet-based method [77]; GPU-based Image reconstruction using the filtered back-projection algorithm implemented in the tomopy library in conjunction with the ASTRA-toolbox [78,79] and application of a circle correction algorithm in the reconstructed domain, provided by the tomopy library [80].
4. Binning the image stacks to size 243 × 243 × 200 voxels using a moving average algorithm to reduce the memory footprint and speed up further processing.
5. Image segmentation is based on a convolutional neural network using the Tensorflow library [81,82].

The image reconstruction process was optimized for image segmentation performance. Filtering was parameterized to favor contrast over sharpness in the final reconstructed images. Image artifact corrections (stripe removal and circle correction) were applied to reduce the heterogeneity of the image quality as much as possible. The motion blur present to varying degrees in most of the acquired CT time-series could not be addressed this way. A convolutional neural network-based image segmentation approach had to be employed to overcome this challenge and enable quantitative data analysis. The reconstructed image stacks were binned from their original dimensions (usually 2016 × 2016 pixels) to 243 × 243 pixels to make the data compatible with the memory limitations of the GPU-accelerated convolutional neural network segmentation and to reduce the permanent storage footprint of the reconstructed CT data. The z-dimension of the image stacks was binned by the same factor used for the *x* and *y* dimensions.

### 2.5 Convolutional neural network-based image segmentation

The reconstructed image stacks were segmented using a convolutional neural network architecture to enable quantitative analysis. The contrast in radiographic densities of the different materials allowed for semantic segmentation [83] into the following classes:

(1) Organic tablet material, consisting of API, disintegrant, lubricant, and, if present, organic filler material (in formulations N33 to N64)
(2) Inorganic tablet filler material (present in formulations N1 to N32)
(3) Background, consisting of varying combinations of air, water, and the tablet holder
(4) Circular mask, artificially introduced during image reconstruction

**Fig. 1:**
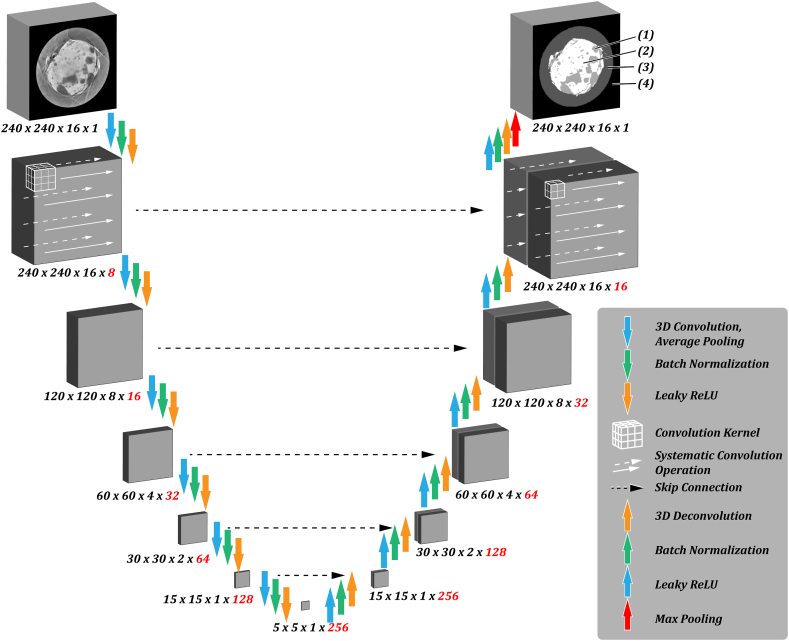
Schematic representation of the U-Net architecture used for image segmentation. Colored arrows indicate sequential computational operations applied to the data. The dimensions of the image data on its way through the network are indicated below each matrix. Feature channels indicated by red numbers are not represented graphically. The convolution kernel and its systematic application to the data are not drawn to scale. Skip connections result in a concatenation in the 4th dimension (visually represented in the 3rd dimension). Segmentation classes are indicated in the output image.

Fig. 1 illustrates these segmentation layers for a tablet that contains an inorganic filler.

Image stacks of tablets that include inorganic filler material in their composition can be segmented into one more class than their purely organic counterparts. Therefore, the reconstruction effort was divided into two subtasks, each using a CNN of identical structure but different amounts of output classes, and each one trained on separate training data.

A CNN with a U-Net architecture [66] was created for the image segmentation task. In general, what sets CNNs apart from other deep learning algorithms is that rather than flattening their input into a vector for further processing, they functionally apply convolution operations to the original matrices of pixel values to extract features from images and thus preserve spatial information. The convolution operation is applied using a kernel, which is systematically shifted by a stride length across the image. Multiple convolution operations are applied in series, and the dimensions of the images are reduced along the way, either by adjusting the stride length to be greater than one or by introducing pooling layers to shrink the images after each convolution operation. Each convolution is usually performed with many different kernels so that each kernel extracts different features of the original image, and subsequent filters specialize even further on extracted aspects of those features. While the images get progressively smaller with each consecutive convolution operation, the number of different kernels applied for operation increases, resulting in more images produced each step. Thus, feature density increases down the chain of convolution operations.

The U-Net architecture is a form of CNN specialized in image segmentation (see Fig. 1). Rather than consisting solely of convolution operations that finally feed into fully connected layers for object classification, the U-Net features a set of deconvolution layers that build the output of the convolution layers back up to a full-sized segmented version of the input image. The deconvolution operations are the functional opposite of the convolution layers. They utilize sets of kernels to expand the input size. The convolution arm and the deconvolution arm are mirrored in function and are interconnected in a way that results in a U-shaped layout, lending the architecture its name. Rather than being connected solely at the lowest level, the U-Net makes full use of its higher-level extracted features by connecting the output of each level of the convolution arm with its corresponding deconvolution operation by concatenating the convolution output with the input of the deconvolution step. These connections are referred to as skip connections.

The network is trained by iteratively adjusting each kernel's values. A set of training data consists of images and their corresponding segmented versions. Each iteration of the training process, an epoch, starts with applying the kernels to the training set. The performance of the current kernel values is assessed according to the resulting output, which is initially completely random. This assessment is compared to the supplied ideal segmentation data, called ground truth data, and is scored using an error function. Image analysis, in general are adjusted toward the greatest expected benefit using an optimization function with the gradient descent method. The performance assessment of the current kernel values is validated on images not involved in the training process to avoid overfitting. The training process is repeated until the best possible network performance is achieved.

Fig. 1 shows the network architecture created to segment the present CT dataset. It comprises six levels of recurring convolution, pooling, batch normalization, and rectification operation blocks on the network's convolution (left) arm. The deconvolution (right) arm inverts the convolution action and reconstructs the data using 3D deconvolution operations. Convolution and deconvolution use 3D kernels due to the three-dimensional structure of the CT images. The images are fed into the network as 3D stacks of 16 × 240 × 240 pixel dimensions. The convolution kernels of size 3 × 3 × 3 are shifted across the data using a stride size of one. A subsequent moving average pooling operation results in dimensionality reduction. A modified rectified linear activation function (leaky ReLU) [84] is used to avoid the vanishing gradient problem [85] and to adjust the output according to Equation (1):

(1) $$h^{\left(i\right)}=\max\left(w^{\left(i\right)T}x,0\right)=\left\{ \begin{array}{c} w^{\left(i\right)T}x,w^{\left(i\right)T}x>0\\ 0.1w^{\left(i\right)T}x,w^{\left(i\right)T}x\leq 0 \end{array}\right.$$

where the activation of a hidden unit $h^{\left(i\right)}$ is given by the value of the previous layer x multiplied by the weight vector $w^{\left(i\right)T}$ for that hidden unit $h^{\left(i\right)}$. To overcome the limitation of standard ReLUs where nodes that start at zero are not adjusted by the optimization algorithm, the ‘leaky ReLU’ function [84] allows for minimal, non-zero gradients even while a node is inactive.

In deep learning, the operation that expands the image on the deconvolution arm is called a deconvolution operation [86], which functionally consists of a convolution operation followed by a transpose operation to expand its size. The network presented in this work uses kernels of size 2 × 2 × 2 for the convolution operations in the righthand side of U-Net.

The last deconvolution step produces a 3D stack in the dimensions of the final image (240 × 240 × 16). Depending on the number of segmentation classes, this penultimate stack has three or four feature channels for purely organic tablets and tablets with inorganic filler, respectively. The activations of the voxels in each feature layer can be considered the probability determined by the U-Net of that voxel belonging to that segmentation class. The segmented image is finally produced by condensing the feature channels into one and assigning segmentation class labels based on which feature channel has the most prominent activation using a max-pooling operation.

### 2.6 Generation of training and validation data

Separate training and validation datasets were created for tablets with organic components (set 1) and organic and inorganic ingredients (set 2). The procedure was the same for both, except set 2 includes one more segmentation class for the inorganic tablet components, i.e., class (2).

The image analysis program Ilastik [86] was used to manually create what the U-Net would then use as ground truth segmentation data for training and validation. That data consisted of 3D reconstructed image stacks and their segmented counterparts of size 240 × 240 × 16, matching the input dimensions of the U-Net. The input dimensions of the U-Net were smaller than those of a single reconstructed CT image (200 × 240 × 240) due to limitations with GPU memory during training. In order for the U-Net training to train on a sufficiently large number of different input image stacks at once, the reconstructed CT image volumes were batched along the z dimension and fed into the U-Net in stacks of size 16. Set 1 consisted of 480 such stacks, and set 2 contained 465 of them. Before CNN training, each image stack was randomly assigned to either the training or the validation datasets at a ratio of three to one. The training stacks were chosen randomly across the different formulations and timepoints.

The training data was generated using Ilastik's manually assisted pixel classification workflow to segment each reconstructed image stack into a mask (class 4), background (class 3), organic tablet (class 1), and, where applicable, inorganic tablet (class 2). The pixel classification workflow in Ilastik involves training a Random Forest classifier, applied manually for each image stack by annotating a subset of four to five evenly spaced images across the stack. The trained classifier was then applied to the entire stack, followed by visual inspection. The manual annotation was iteratively refined until segmentation quality reached a plateau, where further refinements noticeably reduced the segmentation quality in the entire image stack. Due to the variance in image quality between tablet types and time points, this procedure had to be done individually for each formulation and time point, making this task very time-consuming.

For more details on training data generation, see section S2 in the supporting information.

### 2.7 Training of the CNN

The neural networks were trained on an NVIDIA A100 40 GB GPU (Nvidia Corporation, California, USA) using the ADAM [87] optimizer at a constant learning rate of 0.001 and using a sparse categorical cross-entropy [88] error function. For a detailed description of the training process, refer to section S2 of the supplement.

The trained network states (weights and structure) were saved to a file. The complete set of μCT data was then segmented by applying this trained network in a process referred to as inference. The reconstructed μCT image stacks were separated into stacks of 16 × 240 × 240 voxels to match the input dimensions of the U-Net to run the inference. If necessary, the image stacks were zero-padded to make that division possible. The stacks were separated along the z-axis with 4 overlapping voxels to avoid padding operations influencing the inference's performance. The resulting predicted segmentation masks were stitched to remove the overlap. The inference requires much less computational power than the CNN training process and also takes much less time. The trained network states for set 1 and set 2 are available online [74,89].

### 2.8 Data preparation for visual inspection

The reconstructed time-resolved μCT was converted to videos, one for each formulation, to facilitate visual inspection of the dynamic process. The videos’ framerates were adjusted to match the various acquisition framerates, ensuring that the footage represents events in real-time. Consequently, the playback speed of the video accurately reflects the timeline of the experiments. Using masked volumetric image data allowed for removing background layers, reducing the visual clutter, and improving the visual inspection process. The time-resolved volumetric image data were represented as videos by extracting three planes from the original volumetric image. All three planes are cross-sections through the center of the volumetric image, providing top, front, and side views into the tablet as it disintegrates. This way, much of the visual information could be preserved despite the reduction in image dimensionality. The videos are available online [90].

### 2.9 Data analysis

In quantitative analysis, disintegration times and a swelling rates were extracted for disintegrating formulations and swelling formulations respectively. These measures were obtained directly from the segmented image data with a Python script [74]. The script extracts the number of voxels associated with the tablet's solids (organic and inorganic) at every time point for each formulation. The resulting number of voxels was then related to the number of initial voxels for each tablet, thus normalizing the data to the intact dry state of each tablet. Disintegration times were derived for formulations containing the disintegrant croscarmellose sodium or sodium starch glycolate by fitting three-part piecewise functions to that data. For formulations containing hydroxypropyl methylcellulose or polyethylene oxide, a swelling rate was derived by fitting two-part piecewise functions.

Qualitative data analysis collected the subjective formulation performance ratings from the real-time videos. Two human experts in pharmaceutical technology evaluated the videos of the disintegration behavior of each formulation. They were asked to rate each formulation's erosion and swelling intensity separately on a scale of one to ten. This rating scale is a combined measure of how distinctly the disintegration is visually driven by erosion or swelling and the extent of each of those behaviors (one – not very characteristic, not extensive; ten – very characteristic behavior, extensive swelling/erosion).

### 2.10 DOE data response modeling

In factor analysis, a polynomial model was used on the three different responses obtained from the data analysis. Test formulations were prepared according to full factorial design (2∙4∙2^3^) to determine the significance of each formulation component, i.e., the main effects and their interactions on erosion and swelling intensities and the disintegration rate.

The experimental design was done in STAVEX (Aicos AG, Basel, Switzerland) software. The response model analysis was done using R [91] in RStudio [92]. The responses were transformed (y^2^ or y^−2^) where appropriate to yield the best goodness of fit. The responses of formulations containing disintegrants were fitted separately from those containing swelling polymers to individually model the difference between disintegrant choice and swelling polymer choice.

## 3 Results

### 3.1 Image reconstruction

**Fig. 2:**
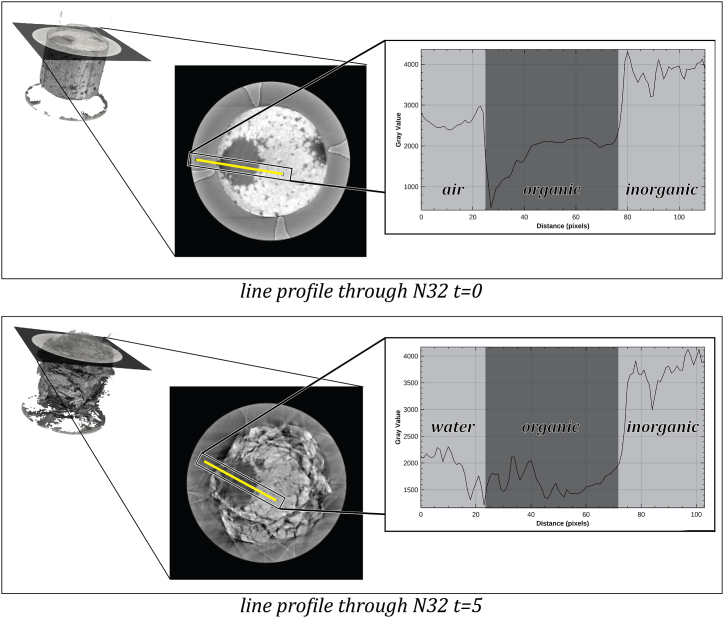
Pixel intensity profiles across tablet layers. Pixel intensities are drawn along a line (yellow bar) across a section of tablet N32 (Supporting information Table S2). Intensity profiles were manually annotated as background (water or air), inorganic- and organic tablet components. Timepoint 0 shows the tablet without water; timepoint 5 shows the same tablet undergoing disintegration in contact with water. The line profiles are located in a similar part of the tablet.

Examples of reconstructed image slices are shown in Fig. 2. The quality of the reconstructed μCT image stacks is very heterogeneous, even though the reconstruction was parameterized to maximize image quality for segmentation in terms of contrast. The image quality of the intact tablets before introducing water was good for all compositions. Only the overall brightness of the tablets and contrast between the different tablet components and the background varied from tablet to tablet due to the varying radiographic densities of the different formulation components. Image artifacts started to appear after the introduction of water. These artifacts’ severity and occurrence frequency differed from one tablet to another at various times.

Furthermore, the image quality correlated with the amount of motion present in a sample, i.e., rapidly disintegrating tablets had poorer image quality. These motion artifacts arise from violating the assumption of a static sample during the acquisition of a single scan, which is crucial for correct CT reconstruction. The presence of artifacts was detrimental to the contrast between different components as well as to image sharpness.

Linear segmentation algorithms turned out to be insufficient to address these challenges. However, it was possible to overcome the problems with image quality and enable image segmentation by applying deep learning-based image segmentation trained on a manually segmented subset of the data, as outlined in the next section.

### 3.2 Performance of the CNN

**Fig. 3:**
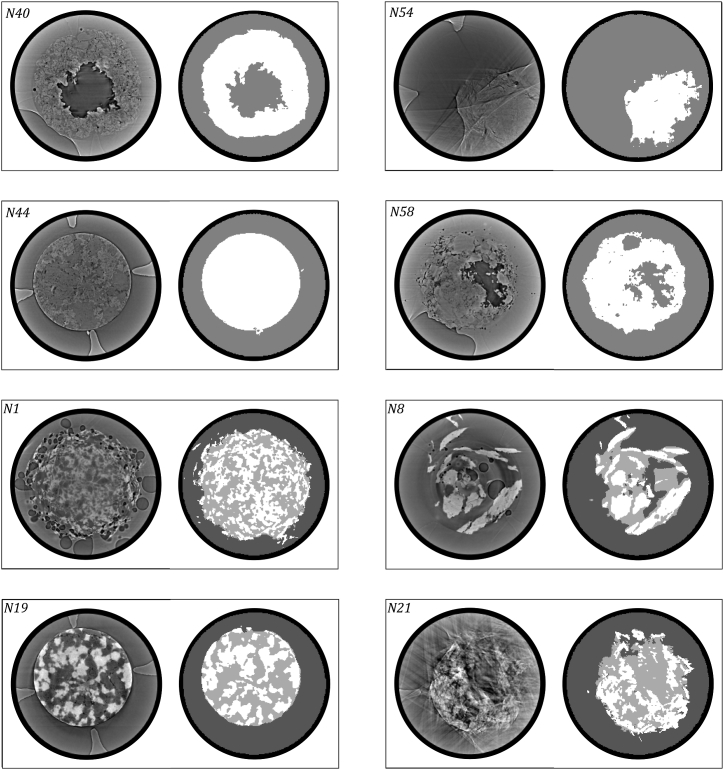
Segmentation performance of the trained U-Net. Formulations N40, N44, N54, and N58 belong to set 1 (3 segmentation classes due to organic filler materials cellulose or mannitol), formulations N1, N8, N19, and N21 belong to set 2 (4 segmentation classes due to inorganic filler materials Fujicalin or FCC). A representative sample of tablet cross-sections at different stages of disintegration is shown. The cross-sections shown for formulations N19 and N44 are at time zero, i.e., in the absence of water. The artificial mask layer (class (4)) is truncated for visibility for all cross-sections. The compositions for all formulations are listed in the supporting information, Table S2.

Fig. 3 shows examples of the segmentation performance of the trained U-Nets for set 1 and set 2, respectively. The segmentation quality is good, based on visual comparison, especially considering some CT scans’ image quality, such as those shown for formulation N54 from set 1 or formulation N21 from set 2. In both cases, it is not easy to distinguish the tablet from the background by eye. Any attempt at segmentation falls firmly in the realm of human subjectivity. Nevertheless, the segmentation created by the trained neural networks holds up to visual analysis. Due to the nature of the reconstructed images, any attempt to segment the entirety of a dataset like this using one consistent ruleset results in errors. However, we can assume this error to be consistent across the entire dataset if using a deep learning-based segmentation, making it possible to quantitatively analyze the disintegration behavior from one timepoint to the next and enabling comparison between the results from different formulations. The output of segmentation methods that need to be run separately on each formulation and timepoint, such as segmentation in Ilastik, would not be consistent enough for such analyses.

The training time was about 5 h per network. Inference, including IO operations, CT image batching, and stitching of the results, took about 25 s per 16 × 240 × 240 image stack. The total inference time for the whole dataset was around 270 h. Optimistic estimates put the time requirement to do the same using manual segmentation in Ilastik at around 2200 h.

A notable weakness of the network trained on data set 1 is a tendency to wrongly label large tablet sections that are very homogeneous in texture and have grey pixels as background. This behavior can be seen for formulation N58 in Fig. 3 and reoccurs sporadically over the entire dataset. The segmentation created by the U-Net trained on data set 2 contains some false positives for the organic tablet class (class (1)) in the surrounding area of detached tablet fragments. This behavior, represented for formulation N1 in Fig. 3, leads to a loss of detail due to the misclassification of gaps and small cracks between tablet fragments.

The dry scans of set 1, seen for formulation N44, are of sufficient quality to differentiate further between the organic formulation components. However, as soon as the water is introduced to the sample, the image quality degrades to a point where this discrimination is no longer possible, as seen for the other images in Fig. 3. Because a segmentation approach that applies to all formulations at all timepoints is desired, set 1 has 3 segmentation classes.

Further details on CNN performance, including performance metrics in the form of confusion matrices, can be found in the supporting information, section S2 and Tables S7 and S8.

### 3.3 Data analysis

**Fig. 4:**
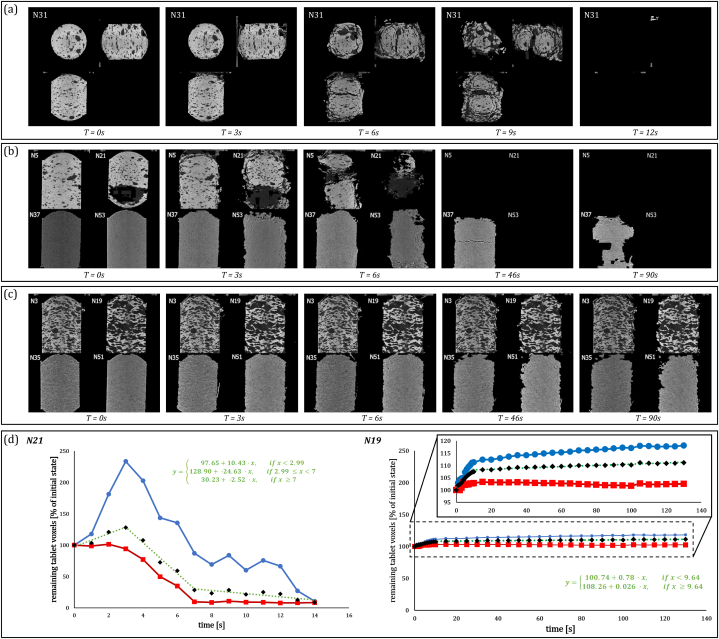
Single frames from the CNN-segmented μCT data visualization videos for various formulations (Table S2). (a) Single frames from the visualization of formulation N31. Cross sections cover the top, side, and front views; time is indicated below the video frame. (b) Single frames for croscarmellose sodium formulations N5, N21, N37, and N53; the filler material is FCC, Fujicalin, cellulose, and mannitol, respectively. (c) Formulations with polyethylene oxide N3, N19, N35, and N51. (d) Change in percentage of voxels associated with organic (blue) and inorganic (red) tablet components and their sum (black) versus time for formulations N21 and N19. Values are normalized to the initial dry tablet state of each component. The piecewise functions used to fit the data are indicated in green.

Video still frames resulting from visualizing the CNN-segmented μCT data in real-time are shown in Fig. 4(a)–(c). They serve to provide insight into the disintegration process of each formulation.

The segmented data was used to remove background pixels from those videos, enhancing visual clarity, especially at advanced stages of disintegration. The consistency of the CNN-based segmentation across the entire dataset thus enables visual analysis and the comparison of the disintegration behavior for all formulations.

The individual videos for each formulation, shown as still frames for formulation N31 in Fig. 4a, were combined like in Fig. 4b and c to compare the disintegration behavior of similar formulations directly.

In these comparison videos, the filler material was observed to strongly impact the effectiveness of disintegrant action. This difference is shown in Fig. 4b for formulations containing croscarmellose sodium, where formulation N37 containing microcrystalline cellulose as the filler material disintegrated slower than the equivalent formulations containing FCC (N5), Fujicalin (N21), and mannitol (N53).

The influence of the filler material could also be observed for swelling formulations. As shown in Fig. 4c, the disintegration of formulations containing polyethylene oxide involved different rates of erosion and crack formation, depending on the filler material (N3 - FCC, N19 - Fujicalin, N35 - microcrystalline cellulose, and N51 - mannitol).

The expert opinion rating of the data was conducted based on the differences observed in the disintegration behavior of different formulations. The supporting information shows the swelling and erosion intensity ratings in Table S9 and Table S10, respectively.

Results from the quantitative rate constant analysis (Fig. 4d) plot the number of tablet voxels versus time for the disintegrating formulation N21. The organic tablet components show a rapid initial increase in volume, likely attributable to the swelling of a disintegrant. Shortly after the initial peak, the organic phase volume decreases rapidly. This decrease coincides with a similar decrease in the non-soluble, non-swelling inorganic tablet components. Such a synchronized decrease in tablet components likely indicates the compact's overall loss of structural integrity, with tablet fragments breaking off and dispersing. The simple linear approximation of the initial and final amounts of tablet components adequately represents the process kinetics. The disintegration rate constants for all formulations containing disintegrants are presented in Table S9.

The swelling kinetics for formulation N19 containing polyethylene oxide are shown in Fig. 4d. In these swelling formulations, the loss of structural integrity could not be observed throughout the experiment. The slow and steady increase in total tablet volume can be attributed to the polymer tablet components’ water-binding capacity. In contrast, the inorganic tablet components slowly decrease over time after an initial fast increase in volume. The swelling rate constants for the formulations containing polyethylene oxide or hydroxypropyl methylcellulose are given in Table S10.

### 3.4 DOE response model analysis

**Fig. 5:**
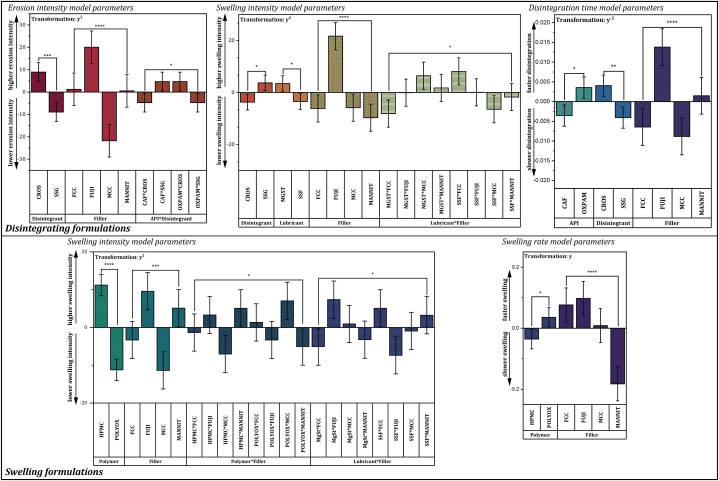
DOE response model factors. Significant parameters of the fitted model are listed for the disintegrating formulations (containing croscarmellose sodium or sodium starch glycolate) and the swelling formulations (containing hydroxypropyl methylcellulose or polyethylene oxide). Levels of statistical significance: * = p < 0.05, ** = p < 0.01, *** = p < 0.001, **** = p < 0.0001. Error bars indicate 95% confidence intervals. Abbreviations: CAF – caffeine, OXPAM –oxantel pamoate, CROS – croscarmellose sodium, SSG – sodium starch glycolate, HPMC – hydroxypropyl methylcellulose, POLYOX – polyethylene oxide, MGST – magnesium stearate, SSF – sodium stearyl fumarate, FCC – functionalized calcium carbonate, FUJI – Fujicalin, MCC – microcrystalline cellulose, MANNIT – mannitol.

The DOE response modeling reveals several interesting insights into the influence of the formulation components on the response variables. All the statistically significant (p < 0.05) factors of the resulting polynomial model and their coefficients are summarized in Fig. 5, and the DOE response parameters used to fit the models are listed in the supporting information, Table S9 and Table S10.

The response model coefficients indicate high significance of the filler material for the disintegration behavior of formulations with disintegrants as well as for swelling formulations. The filler material type is the most statistically significant factor for all responses except for the selling score of swelling formulations, where it is the second most significant factor.

For formulations containing croscarmellose sodium or sodium starch glycolate, the type of disintegrant is a significant factor for all three modeled responses. The interaction of API and disintegrant is an additional significant factor for the erosion intensity score. The swelling intensity score is significantly influenced by the lubricant and the lubricant's interaction with the filler material. The choice of API was a significant factor for the model describing disintegration time.

For swelling formulations based on hydroxypropyl methylcellulose or polyethylene oxide, the choice of polymer was a significant factor for both the swelling intensity as well as the modeled swelling rate. Two interaction factors were additionally significant in the fitted model for swelling intensity, namely the interaction of the swelling polymer and the filler material, and the interaction of lubricant and filler material. The lubricant as a main effect factor was not significant. No model of acceptable fit could be found for the erosion intensity score.

## 4 Discussion

### 4.1 Experimental setup

Synchrotron radiation enabled the acquisition of time-resolved μCT data. The use of a synchrotron facility imposes some restrictions in the experimental setup. The necessity for the sample to rotate during projection acquisition rather than the detector and the X-ray source is such a restriction. The tablets were mounted on a rapidly rotating sample stage on which they should remain as centered and motionless as possible within the frame of reference of the rotating sample stage. Physically restraining the tablet with a tablet holder was a necessary deviation from conventional tablet disintegration experiments. The tablet holder was designed to minimize the contact area between the tablet and holder so that the tablets were under no great strain as they were held in place.

It should also be noted that sample rotation during the acquisition directly influences the direction and the magnitude of the forces acting on each detached tablet fragment by adding a centrifugal term. Therefore, the time required for the tablet to disintegrate under such conditions is possibly reduced. However, for tablets of 1 mm radius and a rotation speed of 360°/s as used for the present dataset, the apparent outward force *F* (F = m·r·ω^2^, where *m* is the mass, *r* is the radius, and *ω* is the angular velocity) resulting from rotation, amounts to approximately 0.4% of the gravitational force, and can thus be considered negligible.

Another necessary compromise between experimental constraints and accurate disintegration simulation was introducing water to the system through a syringe pump above the tablet. The continuous stream of water around the tablet, combined with the rotation of the sample stage, ensured sink conditions while minimizing the X-ray attenuation by the disintegration medium. In addition, degassed ultrapure water was used as the dissolution medium rather than simulated gastric fluid or a physiological buffer to keep bubble formation due to radiolysis [93] during the experiment to a minimum.

### 4.2 Time-resolved μCT

The settings used to acquire the time-resolved μCT data were chosen based on best practices to maximize the image quality of this previously untested experimental setup. The acquisition was not explicitly adapted to best suit a CNN-based image segmentation approach because the need to apply deep learning in data analysis was unknown at the time of acquisition. In retrospect, an increase in acquisition speed at the expense of spatial resolution would have been beneficial. That trade off would have reduced motion artifacts and eliminated the need to reduce image resolution by binning the reconstructed data to a manageable size for deep learning implementation. The impact of reducing the image resolution on the analyses performed in this work, both visual and automated, is assumed to be small. This is because the overall dynamics of the disintegrating tablets were assessed rather than highly localized dynamics in the tablet's microstructures. These analyses rely on the macroscopic features of the individual images, which are preserved after the binning operation. A visual comparison of the image quality of the reconstructed images before and after binning can be seen in the supporting information, Figs. S4 and S5. It is tempting to speculate on the other hand, that future approaches running on more powerful computational hardware or using distributed computing might be able to take full advantage of the high resolution of the raw data and reveal yet hidden details. In contrast to the scoping analysis presented in this work, such focused approaches can simplify the image segmentation task by excluding data below a set quality threshold.

A different strategy for dealing with the computational limitations of the deep learning approach would have been to segment the reconstructed CT data at full scale but in tiny batches. After segmentation, these batches could have been stitched together to retain the full image resolution. The strategy chosen in this work was to segment data that span the entire reconstructed field of view. It was preferable because the segmentation algorithm could work with macroscopic features, leading to better performance despite the reduction in resolution. This strategy was selected based on the current dataset's image quality. We speculate that performing segmentation on small subsets of the reconstructed images would be a much more challenging task. Even human operators would struggle to identify sections of highly enlarged tablet features without seeing the macroscopic features in the full field of view, especially when they are highly blurred.

### 4.3 CNN training and performance

Interestingly, in some cases, the CNN outperformed the training data. Some small misclassifications in the training data are no longer present when the trained network segments the same data. That indicates the CNN training process created a compromise solution suitable for all the data it was trained on. However, there are instances of the trained CNN making mistakes that are not present in the training data. One example is the overeager assignment of the ‘organic tablet material’ (class (1)) label when classifying small cracks in the tablet material, resulting in the loss of small image features in the segmented data.

Overall, the U-Net architecture in the configuration chosen for this work is well-suited for the present segmentation task. The specific network architecture configuration, such as input image stack dimensions, depth of the network, convolution kernel sizes, number of feature channels per layer, and activation rectification, as well as the training parameters such as the optimization function, the loss function, learning rate and stopping parameters, were all derived and refined from a configuration commonly used for image segmentation. The U-Net architecture could likely be further improved by using more sophisticated error- and optimization functions and by testing more combinations of kernel sizes and step sizes. Entirely different network architectures were not explored, given the U-Net architecture's satisfactory performance and relative simplicity.

The trained U-Net rapidly completed its image segmentation task without further user input. Its segmentation performance matched the manual segmentations created in Ilastik regarding accuracy and surpassed them in speed by an order of magnitude. Comparative visual inspection of the segmented data revealed major improvements to the consistency of the segmentation using the U-Net over the manual segmentation. It should be emphasized that the use of deep learning-based image segmentation was practically necessary for the present task due to the size of the dataset. Even if Ilastik's machine learning-assisted manual segmentation approach could deliver consistent results over different tablet formulations and time points, such a manual approach would take too much time to apply to the entire dataset to be feasible. Furthermore, we are convinced that any conventional, non-machine learning-based algorithmic approach to image segmentation cannot cope with the heterogeneity of the reconstructed image data quality of the present dataset.

### 4.4 Influence of formulation components on disintegration behavior

The modeling of the responses to the visual and the quantitative data analysis revealed differing impacts of specific formulation components on disintegration behavior.

The solubility of API, for example, played a significant role in the disintegration time of the formulations containing the super disintegrants croscarmellose sodium or sodium starch glycolate. Due to its moderate water solubility, caffeine has significantly increased a formulation's disintegration time. On the other hand, oxantel pamoate significantly reduced the disintegration time despite the compound's very poor aqueous solubility. This counterintuitive behavior is consistent with published experimental data, for example, of J. von Orelli, who found tablets containing caffeine to have slower disintegration times than the formulation of poorly soluble proquazone [94]. The disintegrant type also played a statistically significant role in the model for disintegration time. The presence of croscarmellose sodium sped up disintegration in comparison to sodium starch glycolate, indicating that the former acts more rapidly than the latter, a difference that has been reported in the literature [95]. Notably, the type of filler material in the formulation had the most significant impact on the disintegration time constant of all the investigated factors. The inorganic filler material Fujicalin reduced the disintegration time, while FCC, though chemically similar [96,97], slowed down the disintegration slightly. The reason for that is a difference in the pore size distribution of the compacts made with FCC and Fujicalin. Pores in the FCC compacts are smaller and thus have a higher hydrodynamic resistance for disintegration medium. Mannitol as a filler material slightly decreased the disintegration time, whereas microcrystalline cellulose (MCC) increased the disintegration time significantly. This finding, reported in the literature [98], is corroborated by the video data of formulations differing only in filler material, as shown in Fig. 4b, where formulations containing MCC disintegrate noticeably slower than those with non-swelling constituents. Further supporting this finding, the presence of MCC in a formulation significantly reduced the scores in both the erosion and swelling intensity response models, indicating an overall inhibitory effect of MCC on all disintegration activity in the formulations. These results may be seen as counterintuitive as MCC is commonly thought to help disintegration thanks to its swelling, however it binds water and, therefore, reduces the rate at which liquid can reach the superdisintegrant. Our findings support the ‘competition for water’ theory presented in a publication by Ekmekciyan et al. [99] in which they propose the formulation component's competition for water as a way of understanding their observations on differing disintegration performance when comparing formulations with soluble and insoluble filler material. Based on our observations this theory seems to hold not only for the water binding properties of the filler material, but also for highly soluble actives. The choice of API had a significant impact on disintegrant time and on erosion intensity by its interaction with the disintegrant.

The models on erosion and swelling intensity of disintegrating tablets show opposite impacts of each of the two disintegrants. Croscarmellose sodium increases the erosion intensity while decreasing the swelling intensity of a formulation, whereas sodium starch glycolate has the opposite effect. Our finding is in agreement with sodium starch glycolate's reported larger swelling capacity than croscarmellose sodium's [100]. The filler material is again the most significant factor in both models. Fujicalin significantly increased erosion and swelling intensity, leading us to propose that it facilitated disintegrant action in general, possibly by efficient water delivery through its porous meshwork [2,4]. Lubricant type was a significant parameter for the swelling intensity model, both as the main effect and by its interaction with the filler material type. Thus highlighting that care should be taken when choosing the combination of the lubricant and the disintegrant for a given formulation. Magnesium stearate increased the swelling intensity score compared to sodium stearyl fumarate partially due to their different size differences but more likely owing to their different aqueous solubility [101]. As the swelling is promoted by the liquid imbibition, it is sensitive to the surface-liquid contact interface properties, i.e., how hydrophobic the surface is. Both lubricants make the surface more hydrophobic. We however assume that their interactions with the lamellar shell of the comparatively small FCC particles (mean diameter: 7.28 μm) [96] are different than their interactions with the much smoother surface of the larger particles of the Fujicalin (mean diameter: 120 μm) [102]. No model of adequate fit could be found for the erosion intensity score of the swelling formulations containing hydroxypropyl methylcellulose or polyethylene oxide. That is because the formulations don't erode in the timescales covered by the μCT acquisitions. Their behavior can, therefore, almost exclusively be described by the swelling score rather than by a combination of swelling and erosion, like for the formulations containing disintegrants.

The choice of polymer is the most significant parameter in the swelling intensity score model of the swelling formulations. Hydroxypropyl methylcellulose in a formulation increased this score, while polyethylene oxide lowered it. The linear polymer structure of PEO suggests fast water binding ability but also the formation of weaker, erodible gels [101,103]. Therefore, in the presence of factors facilitating erosion of the polyethylene oxide gel, the swelling intensity is not as prominent, as seen from this study's experimental data. The filler materials affect the swelling intensity the same way as discussed for the disintegrating formulations, with Fujicalin significantly increasing the swelling intensity score and microcrystalline cellulose significantly lowering it. As with the disintegrating formulations, we suggest this difference comes from the different interactions of these materials with water, with Fujicalin passing it on to the swelling polymers by wicking and the microcrystalline cellulose competing for the water by swelling. The presence of mannitol increased the swelling intensity slightly, and FCC did not significantly influence the score. In addition to this main effect, the filler materials had a statistically significant influence on the swelling score in their interactions with the swelling polymer and the lubricant. The interaction of microcrystalline cellulose with hydroxypropyl methylcellulose significantly decreased the swelling score, while its interaction with polyethylene oxide increased it. We propose competition for water by the microcrystalline cellulose to be the mechanism for this behavior, which reduces the amount of water available for the swelling polymer, which in the case of hydroxypropyl methylcellulose seems detrimental to its swelling action. In contrast, the polyethylene oxide is less sensitive to decreased water influx and thus comparatively benefits from this interaction. Amongst the interactions of the lubricant with the filler material, Fujicalin produces the most significant effect. In combination with magnesium stearate, the interaction increases the swelling intensity, whereas with sodium stearyl fumarate, the interaction decreases the swelling score. We suggest our finding on the interaction of Fujicalin with magnesium stearate to reflect the general tendency of magnesium stearate to slow down the disintegration of formulations. In contrast, sodium stearyl fumarate is less hydrophobic and thus affects tablet dissolution less [101].

The model for the swelling rate contains polymer type and filler type as significant parameters. The influence of the polymer type is in line with the observations made for the selling intensity score. Polyethylene oxide increased the swelling rate as compared to hydroxypropyl methylcellulose. The filler materials' influence on the swelling rate differed: FCC increased the rate, whereas mannitol decreased it. The influence of Fujicalin increased the swelling rate analogously to the swelling intensity score. We hypothesize the swelling rate measure to be closer to reality, as it is based on objective automated analysis of the whole 3D image sequence. We expect that the differences in the swelling intensity score are caused by the difficulty of the data visualization to demonstrate the dynamics of the whole 3D image. The orthogonal 2D image sequences extracted from the image volume center likely don't fully capture the swelling behavior of certain formulations.

Our findings from direct visual analysis of disintegration dynamics align well with the automatically extracted objective measures of swelling intensity and disintegration time. The fitted models are in agreement with the known effects and interactions of the formulation components obtained by indirect measurements. Our numerous observations of the micromechanics governing the disintegration process warrant deeper, more focused exploration. We hope the exemplary analyses presented in this work enable further research into the data and follow-up experiments.

## 5 Conclusion

This study combines deep-learning-based image analysis with time-resolved X-ray microtomography imaging to gain direct insight into the dynamic changes of the internal microstructures of pharmaceutical tablets undergoing disintegration. A broad range of relevant formulations of mini-tablets created according to a DOE, each disintegrating at different speeds, was included in the analysis. Their disintegration behavior was visualized and analyzed qualitatively as well as quantitatively.

We adapted the tablet disintegration experiment to the challenging setting of synchrotron radiation-based tomographic microscopy by creating a custom 3D-printed tablet holder which enabled continuous μCT acquisition of disintegrating tablets - in our experiments at a maximum rate of two complete CT acquisitions per second, each requiring the acquisition of 500 high-resolution projection images.

We created a data processing pipeline to reconstruct, segment, visualize, and quantify the large μCT image dataset. The proposed pipeline is an automated workflow that uses a convolutional neural network to segment the large amount of heterogeneous image data generated by the time-resolved μCT approach.

The segmented data allowed us to create comprehensive, real-time videos of the tablets’ internal structure change during disintegration, providing an overview of the dataset and enabling visual investigation of the differences in disintegration dynamics between the formulations. The segmentation also enabled quantification of swelling rates and disintegration times, each automatically extracted from the data. We use linear regression response modeling to assess the impact of the formulation components and their interactions on these quantitative aspects as well as on qualitative observations based on the visualization. This analysis allowed us to relate our findings to existing research.

In addition to the software code and example data, we publish the trained U-Net and the dataset used in its training and validation. We expect our methodology to benefit the research of other pharmaceutical processes, for example, by applying it to tablet compaction or drug-loading into porous carriers. Using the analysis methods presented in this work, we expect our data to enable researchers to test their hypotheses on the tablet disintegration mechanism by visual inspection and by analyzing disintegration parameters. We thus help to bridge the gap between theories of the disintegration mechanism, that are based on indirect measurements, and a fundamental understanding of the physicochemical phenomena governing this complex process. The data we have acquired and processed will make it possible to verify and refine in silico disintegration simulation experimentally and will support rational pharmaceutical formulation design.

## Data availability

Binned reconstructed CT image stacks and segmented counterparts are available for all tablets, separated by tablet formulation [89], along with the trained neural network states and the annotated data used to train the networks. Full-resolution projection raw data is available on request.

All Code used in the reconstruction, segmentation, and analysis of the μCT data is available on GitHub [74], along with the trained neural networks used for image segmentation. Real-time videos showing the disintegration of each tablet formulation are also available [90].

## CRediT authorship contribution statement

**Samuel Waldner:** Writing – review & editing, Writing – original draft, Visualization, Validation, Software, Methodology, Formal analysis, Data curation, Conceptualization. **Erwin Wendelspiess:** Methodology, Investigation, Data curation. **Pascal Detampel:** Writing – review & editing, Resources, Data curation. **Christian M. Schlepütz:** Writing – review & editing, Writing – original draft, Validation, Resources, Methodology, Investigation, Funding acquisition, Data curation. **Jörg Huwyler:** Writing – review & editing, Validation, Resources, Project administration, Funding acquisition. **Maxim Puchkov:** Writing – review & editing, Writing – original draft, Validation, Supervision, Software, Project administration, Methodology, Investigation, Funding acquisition, Formal analysis, Data curation, Conceptualization.

## Declaration of competing interest

The authors declare that they have no known competing financial interests or personal relationships that could have appeared to influence the work reported in this paper.
