# Supplementary material for: Advanced analysis of disintegrating pharmaceutical compacts using deep learning-based segmentation of time-resolved micro-tomography images
Source: Heliyon. 2024 Feb 12;10(4):e26025. doi: 10.1016/j.heliyon.2024.e26025 (PMC10878950; doi:10.1016/j.heliyon.2024.e26025)
Supplement: Multimedia component 1 [file mmc1.docx]

## Supporting information

Table S1: Design of experiments reference for tablet composition – factors and levels. The super disintegrant polymer level includes AcDiSol and SSG, and the gel-forming polymer level includes HPMC and POLYOX. The DOE results in a blocked qualitative structure (2^3^x2x4)

| **Factor** | **Type** | **Levels** | |
| --- | --- | --- | --- |
| API | Qualitative | (-) Oxpam | |
|  |  | (+) Caffeine | |
| Lubricant | Qualitative | (-) SSF | |
|  |  | (+) MgSt | |
| Polymer class | Qualitative | (-) Superdisintegrant | |
|  |  | (+) Gel forming Polymer | |
| Filler | Qualitative | (-2) FCC | |
|  |  | (-1) FUJI | |
|  |  | (+1) CELL | |
|  |  | (+2) MANNIT | |
| Polymer substance | Qualitative | (-) HPMC | (-) AcDiSol |
|  |  | (+) POLYOX | (+) SSG |

Table S2: Design of experiments for tablet composition – Experimental conditions

| **Formulation Name** | **API** | **Lubricant** | **Polymer class** | **Filler** | **Polymer** |
| --- | --- | --- | --- | --- | --- |
| N1 | Caffeine | MgSt | Gel former | FCC | HPMC |
| N2 | Oxpam | MgSt | Gel former | FCC | HPMC |
| N3 | Caffeine | MgSt | Gel former | FCC | POLYOX |
| N4 | Oxpam | MgSt | Gel former | FCC | POLYOX |
| N5 | Caffeine | MgSt | Disintegrant | FCC | Croscarmellose-Na |
| N6 | Oxpam | MgSt | Disintegrant | FCC | Croscarmellose-Na |
| N7 | Caffeine | MgSt | Disintegrant | FCC | SSG |
| N8 | Oxpam | MgSt | Disintegrant | FCC | SSG |
| N9 | Caffeine | SSF | Gel former | FCC | HPMC |
| N10 | Oxpam | SSF | Gel former | FCC | HPMC |
| N11 | Caffeine | SSF | Gel former | FCC | POLYOX |
| N12 | Oxpam | SSF | Gel former | FCC | POLYOX |
| N13 | Caffeine | SSF | Disintegrant | FCC | Croscarmellose-Na |
| N14 | Oxpam | SSF | Disintegrant | FCC | Croscarmellose-Na |
| N15 | Caffeine | SSF | Disintegrant | FCC | SSG |
| N16 | Oxpam | SSF | Disintegrant | FCC | SSG |
| N17 | Caffeine | MgSt | Gel former | Fujicalin | HPMC |
| N18 | Oxpam | MgSt | Gel former | Fujicalin | HPMC |
| N19 | Caffeine | MgSt | Gel former | Fujicalin | POLYOX |
| N20 | Oxpam | MgSt | Gel former | Fujicalin | POLYOX |
| N21 | Caffeine | MgSt | Disintegrant | Fujicalin | Croscarmellose-Na |
| N22 | Oxpam | MgSt | Disintegrant | Fujicalin | Croscarmellose-Na |
| N23 | Caffeine | MgSt | Disintegrant | Fujicalin | SSG |
| N24 | Oxpam | MgSt | Disintegrant | Fujicalin | SSG |
| N25 | Caffeine | SSF | Gel former | Fujicalin | HPMC |
| N26 | Oxpam | SSF | Gel former | Fujicalin | HPMC |
| N27 | Caffeine | SSF | Gel former | Fujicalin | POLYOX |
| N28 | Oxpam | SSF | Gel former | Fujicalin | POLYOX |
| N29 | Caffeine | SSF | Disintegrant | Fujicalin | Croscarmellose-Na |
| N30 | Oxpam | SSF | Disintegrant | Fujicalin | Croscarmellose-Na |
| N31 | Caffeine | SSF | Disintegrant | Fujicalin | SSG |
| N32 | Oxpam | SSF | Disintegrant | Fujicalin | SSG |
| N33 | Caffeine | MgSt | Gel former | MCC | HPMC |
| N34 | Oxpam | MgSt | Gel former | MCC | HPMC |
| N35 | Caffeine | MgSt | Gel former | MCC | POLYOX |
| N36 | Oxpam | MgSt | Gel former | MCC | POLYOX |
| N37 | Caffeine | MgSt | Disintegrant | MCC | Croscarmellose-Na |
| N83 | Oxpam | MgSt | Disintegrant | MCC | Croscarmellose-Na |
| N93 | Caffeine | MgSt | Disintegrant | MCC | SSG |
| N40 | Oxpam | MgSt | Disintegrant | MCC | SSG |
| N41 | Caffeine | SSF | Gel former | MCC | HPMC |
| N42 | Oxpam | SSF | Gel former | MCC | HPMC |
| N43 | Caffeine | SSF | Gel former | MCC | POLYOX |
| N44 | Oxpam | SSF | Gel former | MCC | POLYOX |
| N45 | Caffeine | SSF | Disintegrant | MCC | Croscarmellose-Na |
| N46 | Oxpam | SSF | Disintegrant | MCC | Croscarmellose-Na |
| N47 | Caffeine | SSF | Disintegrant | MCC | SSG |
| N48 | Oxpam | SSF | Disintegrant | MCC | SSG |
| N49 | Caffeine | MgSt | Gel former | Mannitol | HPMC |
| N50 | Oxpam | MgSt | Gel former | Mannitol | HPMC |
| N51 | Caffeine | MgSt | Gel former | Mannitol | POLYOX |
| N52 | Oxpam | MgSt | Gel former | Mannitol | POLYOX |
| N53 | Caffeine | MgSt | Disintegrant | Mannitol | Croscarmellose-Na |
| N54 | Oxpam | MgSt | Disintegrant | Mannitol | Croscarmellose-Na |
| N55 | Caffeine | MgSt | Disintegrant | Mannitol | SSG |
| N56 | Oxpam | MgSt | Disintegrant | Mannitol | SSG |
| N57 | Caffeine | SSF | Gel former | Mannitol | HPMC |
| N58 | Oxpam | SSF | Gel former | Mannitol | HPMC |
| N59 | Caffeine | SSF | Gel former | Mannitol | POLYOX |
| N60 | Oxpam | SSF | Gel former | Mannitol | POLYOX |
| N61 | Caffeine | SSF | Disintegrant | Mannitol | Croscarmellose-Na |
| N62 | Oxpam | SSF | Disintegrant | Mannitol | Croscarmellose-Na |
| N63 | Caffeine | SSF | Disintegrant | Mannitol | SSG |
| N64 | Oxpam | SSF | Disintegrant | Mannitol | SSG |

Table S3: Compaction forces and compression energies for the different compact formulations recorded by the Styl'One tablet press.

| **Name** | **Upper punch compaction force [kN]** | **Lower punch compaction force [kN]** | **Compression energy [J]** |
| --- | --- | --- | --- |
| N1 | 2.6 | 5.5 | 3.5 |
| N2 | 2.7 | 5.4 | 3.4 |
| N3 | 2.6 | 5.5 | 3.3 |
| N4 | 2.7 | 5.5 | 3.2 |
| N5 | 3.7 | 5.5 | 3.9 |
| N6 | 3.6 | 5.4 | 3.9 |
| N7 | 3.5 | 5.4 | 4.0 |
| N8 | 3.3 | 5.5 | 4.1 |
| N9 | 2.5 | 5.4 | 3.2 |
| N10 | 2.5 | 5.5 | 3.4 |
| N11 | 2.7 | 5.5 | 3.1 |
| N12 | 2.5 | 5.5 | 3.2 |
| N13 | 3.6 | 5.5 | 3.8 |
| N14 | 3.5 | 5.5 | 3.8 |
| N15 | 3.5 | 5.5 | 3.7 |
| N16 | 3.3 | 5.5 | 3.8 |
| N17 | 4.2 | 5.7 | 1.8 |
| N18 | 4.6 | 6.0 | 1.4 |
| N19 | 4.4 | 6.1 | 1.5 |
| N20 | 4.5 | 6.1 | 1.4 |
| N21 | 4.5 | 5.8 | 2.0 |
| N22 | 4.8 | 5.9 | 1.7 |
| N23 | 4.5 | 5.8 | 2.0 |
| N24 | 4.5 | 5.7 | 2.0 |
| N25 | 4.2 | 5.8 | 1.8 |
| N26 | 4.6 | 6.0 | 1.4 |
| N27 | 4.2 | 5.8 | 1.5 |
| N28 | 4.3 | 6.0 | 1.6 |
| N29 | 4.6 | 5.8 | 1.9 |
| N30 | 4.7 | 5.8 | 1.8 |
| N31 | 4.5 | 5.8 | 2.0 |
| N32 | 4.6 | 5.9 | 1.9 |
| N33 | 4.4 | 6.1 | 1.9 |
| N34 | 4.7 | 6.2 | 1.4 |
| N35 | 4.4 | 6.2 | 1.5 |
| N36 | 4.5 | 6.3 | 1.4 |
| N37 | 4.2 | 6.0 | 2.4 |
| N38 | 4.5 | 6.0 | 1.8 |
| N39 | 3.9 | 5.8 | 2.5 |
| N40 | 4.3 | 6.0 | 2.0 |
| N41 | 4.3 | 6.0 | 1.9 |
| N42 | 4.3 | 6.0 | 1.6 |
| N43 | 4.3 | 6.2 | 1.6 |
| N44 | 4.6 | 6.4 | 1.3 |
| N45 | 4.3 | 5.9 | 2.1 |
| N46 | 4.5 | 6.0 | 1.9 |
| N47 | 4.2 | 5.8 | 2.2 |
| N48 | 4.3 | 5.9 | 2.2 |
| N49 | 3.7 | 5.8 | 1.7 |
| N50 | 4.1 | 6.0 | 1.4 |
| N51 | 3.4 | 5.7 | 1.6 |
| N52 | 3.6 | 5.9 | 1.4 |
| N53 | 3.1 | 5.6 | 2.1 |
| N54 | 3.3 | 5.7 | 2.0 |
| N55 | 3.0 | 5.5 | 2.0 |
| N56 | 3.2 | 5.6 | 2.0 |
| N57 | 3.5 | 5.6 | 1.8 |
| N58 | 3.7 | 5.8 | 1.6 |
| N59 | 3.6 | 5.8 | 1.5 |
| N60 | 3.8 | 5.8 | 1.3 |
| N61 | 3.4 | 5.6 | 2.1 |
| N62 | 3.8 | 5.7 | 1.6 |
| N63 | 3.5 | 5.7 | 1.9 |
| N64 | 3.9 | 5.7 | 1.5 |

1. Analysis of mini-tablets

Average tablet weight, diameter, and thickness were measured upon tablet compression. Tablet volumes were calculated based on measured thickness, diameter, and the known tablet geometry according to the following equations 1-3:

|  | $V_{cap}=\frac{\pi}{6}*h*(3r^{2}+h_{cap}^{2})$ | (1) |
| --- | --- | --- |
|  | $V_{cylinder}=\pi*r^{2}*h_{cylinder}$ | (2) |
|  | $V_{tablet}=V_{cylinder}+2*V_{cap}$ | (3) |

Where *V_tablet_* is the total tablet volume, *V_cap_* is the cap volume, *V_cylinder_* is the volume of the cylindrical part of the tablet, *h_cap_* is the cap height, *h_cylinder_* is the height of the cylinder, and *r* is the tablet radius.

The porosity for each tablet could be calculated if combined with the measured mass and true density information

True densities of all formulation components are listed in Table S4 in the supporting information. Tablet dimensions such as weights, densities, and porosity values calculated from those measured quantities are listed in Table S5.


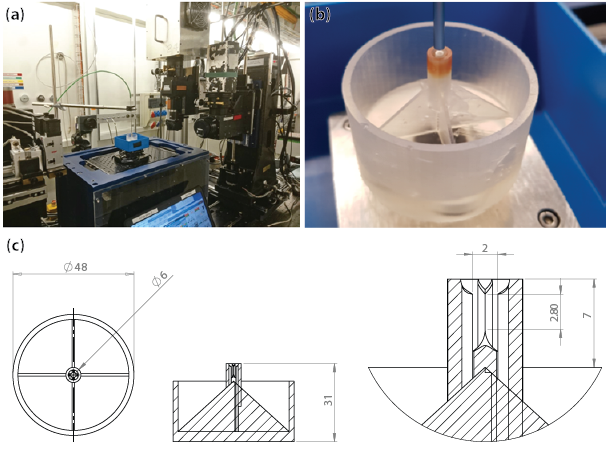


Figure S1: Experimental setup at the SLS TOMCAT beamline. (a) shows the optics and the sample stage, including the tube leading to the syringe pump. (b) shows a close-up of the 3D-printed tablet holder with the syringe pump outlet above it. (c) Technical drawings of the tablet holder with dimensions given in mm.

Table S4: True density values for all formulation components measured by helium pycnometry.

| **Formulation component** | **True density [g·cm^-3^]** |
| --- | --- |
| Caffeine | 1.473 |
| Oxantel pamoate | 1.420 |
| Croscarmellose sodium | 1.473 |
| Sodium starch glycolate | 1.495 |
| Hydroxypropyl methylcellulose | 1.296 |
| Polyethylene oxide | 1.248 |
| Magnesium stearate | 1.094 |
| Sodium stearyl fumarate | 1.281 |
| Functionalized calcium carbonate | 3.087 |
| Fujicalin | 2.940 |
| Mannitol | 1.518 |
| Cellulose | 1.578 |

Table S5: Tablet dimensions. Height and weight were measured directly, true densities of tablet components were measured and added up according to tablet composition, diameter is known, volume, density, and porosity were calculated based on the other known parameters.

|  | **Height** [mm] (n=6) | **Diameter** [mm] | **Weight per tablet** [mg] (n=6) | **Volume** [mm^3^] | **Density** [mg*mm^-3^] | **True density** [mg*mm^-3^] | **Porosity** [%] |
| --- | --- | --- | --- | --- | --- | --- | --- |
| N1 | 4.55 ± 0.01 | 2.00 ± 0.01 | 15.34 ± 0.31 | 13.05 | 1.1760 | 1.8174 | 35.29 |
| N2 | 4.90 ± 0.02 | 2.00 ± 0.01 | 17.75 ± 0.29 | 14.15 | 1.2548 | 1.8090 | 30.64 |
| N3 | 4.46 ± 0.01 | 2.00 ± 0.01 | 16.88 ± 0.41 | 12.76 | 1.3222 | 1.7789 | 25.67 |
| N4 | 4.38 ± 0.02 | 2.00 ± 0.01 | 16.28 ± 0.43 | 12.51 | 1.3015 | 1.7709 | 26.51 |
| N5 | 3.47 ± 0.02 | 2.00 ± 0.01 | 14.92 ± 0.35 | 9.65 | 1.5453 | 2.6300 | 41.24 |
| N6 | 3.50 ± 0.02 | 2.00 ± 0.01 | 13.35 ± 0.42 | 9.75 | 1.3696 | 2.6126 | 47.57 |
| N7 | 3.61 ± 0.02 | 2.00 ± 0.01 | 15.35 ± 0.29 | 10.09 | 1.5209 | 2.6204 | 41.96 |
| N8 | 3.59 ± 0.01 | 2.00 ± 0.01 | 15.02 ± 0.32 | 10.03 | 1.4972 | 2.6031 | 42.48 |
| N9 | 5.00 ± 0.01 | 2.00 ± 0.01 | 18.41 ± 0.44 | 14.46 | 1.2731 | 1.8263 | 30.29 |
| N10 | 5.05 ± 0.01 | 2.00 ± 0.01 | 18.38 ± 0.20 | 14.62 | 1.2577 | 1.8178 | 30.81 |
| N11 | 4.42 ± 0.02 | 2.00 ± 0.01 | 16.70 ± 0.24 | 12.64 | 1.3215 | 1.7874 | 26.07 |
| N12 | 4.57 ± 0.01 | 2.00 ± 0.01 | 18.67 ± 0.33 | 13.11 | 1.4240 | 1.7793 | 19.97 |
| N13 | 3.49 ± 0.02 | 2.00 ± 0.01 | 14.86 ± 0.26 | 9.72 | 1.5293 | 2.6487 | 42.26 |
| N14 | 3.44 ± 0.01 | 2.00 ± 0.01 | 14.21 ± 0.39 | 9.56 | 1.4865 | 2.6310 | 43.50 |
| N15 | 3.44 ± 0.01 | 2.00 ± 0.01 | 14.67 ± 0.32 | 9.56 | 1.5344 | 2.6389 | 41.85 |
| N16 | 3.46 ± 0.01 | 2.00 ± 0.01 | 14.33 ± 0.21 | 9.62 | 1.4897 | 2.6214 | 43.17 |
| N17 | 3.16 ± 0.02 | 2.00 ± 0.01 | 11.73 ± 0.44 | 8.68 | 1.3510 | 1.7921 | 24.61 |
| N18 | 2.67 ± 0.01 | 2.00 ± 0.01 | 9.66 ± 0.32 | 7.14 | 1.3528 | 1.7840 | 24.17 |
| N19 | 3.01 ± 0.02 | 2.00 ± 0.01 | 11.23 ± 0.38 | 8.21 | 1.3676 | 1.7547 | 22.06 |
| N20 | 2.72 ± 0.02 | 2.00 ± 0.01 | 10.03 ± 0.26 | 7.30 | 1.3739 | 1.7469 | 21.35 |
| N21 | 2.75 ± 0.02 | 2.00 ± 0.01 | 11.88 ± 0.44 | 7.39 | 1.6078 | 2.5382 | 36.65 |
| N22 | 2.33 ± 0.01 | 2.00 ± 0.01 | 9.80 ± 0.42 | 6.07 | 1.6141 | 2.5220 | 36.00 |
| N23 | 2.80 ± 0.02 | 2.00 ± 0.01 | 12.43 ± 0.38 | 7.55 | 1.6461 | 2.5293 | 34.92 |
| N24 | 2.64 ± 0.02 | 2.00 ± 0.01 | 11.28 ± 0.22 | 7.05 | 1.6015 | 2.5131 | 36.27 |
| N25 | 3.10 ± 0.01 | 2.00 ± 0.01 | 11.63 ± 0.39 | 8.49 | 1.3692 | 1.8007 | 23.96 |
| N26 | 2.65 ± 0.01 | 2.00 ± 0.01 | 9.38 ± 0.33 | 7.08 | 1.3248 | 1.7925 | 26.10 |
| N27 | 2.91 ± 0.02 | 2.00 ± 0.01 | 10.93 ± 0.23 | 7.89 | 1.3851 | 1.7629 | 21.43 |
| N28 | 2.93 ± 0.01 | 2.00 ± 0.01 | 10.88 ± 0.26 | 7.96 | 1.3668 | 1.7551 | 22.12 |
| N29 | 2.55 ± 0.02 | 2.00 ± 0.01 | 11.18 ± 0.39 | 6.76 | 1.6537 | 2.5556 | 35.29 |
| N30 | 2.40 ± 0.02 | 2.00 ± 0.01 | 10.19 ± 0.40 | 6.29 | 1.6200 | 2.5391 | 36.20 |
| N31 | 2.64 ± 0.02 | 2.00 ± 0.01 | 11.70 ± 0.26 | 7.05 | 1.6607 | 2.5465 | 34.79 |
| N32 | 2.50 ± 0.01 | 2.00 ± 0.01 | 10.59 ± 0.33 | 6.61 | 1.6035 | 2.5302 | 36.63 |
| N33 | 3.03 ± 0.01 | 2.00 ± 0.01 | 10.30 ± 0.26 | 8.27 | 1.2454 | 1.4306 | 12.95 |
| N34 | 2.84 ± 0.02 | 2.00 ± 0.01 | 9.27 ± 0.40 | 7.67 | 1.2076 | 1.4254 | 15.28 |
| N35 | 3.03 ± 0.01 | 2.00 ± 0.01 | 10.33 ± 0.41 | 8.27 | 1.2484 | 1.4066 | 11.25 |
| N36 | 2.86 ± 0.02 | 2.00 ± 0.01 | 9.64 ± 0.28 | 7.74 | 1.2463 | 1.4016 | 11.09 |
| N37 | 3.10 ± 0.01 | 2.00 ± 0.01 | 11.64 ± 0.34 | 8.49 | 1.3712 | 1.5536 | 11.74 |
| N38 | 2.70 ± 0.02 | 2.00 ± 0.01 | 9.79 ± 0.33 | 7.23 | 1.3536 | 1.5475 | 12.53 |
| N39 | 3.33 ± 0.01 | 2.00 ± 0.01 | 12.68 ± 0.27 | 9.21 | 1.3758 | 1.5503 | 11.25 |
| N40 | 2.88 ± 0.01 | 2.00 ± 0.01 | 10.63 ± 0.23 | 7.80 | 1.3623 | 1.5442 | 11.78 |
| N41 | 3.11 ± 0.01 | 2.00 ± 0.01 | 10.48 ± 0.25 | 8.52 | 1.2302 | 1.4361 | 14.34 |
| N42 | 2.95 ± 0.01 | 2.00 ± 0.01 | 9.75 ± 0.34 | 8.02 | 1.2158 | 1.4309 | 15.03 |
| N43 | 2.99 ± 0.01 | 2.00 ± 0.01 | 10.15 ± 0.34 | 8.14 | 1.2462 | 1.4120 | 11.74 |
| N44 | 2.78 ± 0.02 | 2.00 ± 0.01 | 9.29 ± 0.31 | 7.49 | 1.2414 | 1.4069 | 11.77 |
| N45 | 2.80 ± 0.01 | 2.00 ± 0.01 | 10.41 ± 0.42 | 7.55 | 1.3790 | 1.5601 | 11.61 |
| N46 | 2.61 ± 0.02 | 2.00 ± 0.01 | 9.52 ± 0.44 | 6.95 | 1.3691 | 1.5540 | 11.90 |
| N47 | 2.90 ± 0.01 | 2.00 ± 0.01 | 10.92 ± 0.20 | 7.86 | 1.3885 | 1.5567 | 10.80 |
| N48 | 2.61 ± 0.02 | 2.00 ± 0.01 | 9.71 ± 0.43 | 6.95 | 1.3967 | 1.5506 | 9.93 |
| N49 | 3.70 ± 0.01 | 2.00 ± 0.01 | 12.21 ± 0.30 | 10.38 | 1.1767 | 1.4067 | 16.36 |
| N50 | 3.17 ± 0.01 | 2.00 ± 0.01 | 10.25 ± 0.31 | 8.71 | 1.1768 | 1.4017 | 16.05 |
| N51 | 3.63 ± 0.02 | 2.00 ± 0.01 | 12.03 ± 0.25 | 10.16 | 1.1849 | 1.3836 | 14.36 |
| N52 | 3.42 ± 0.02 | 2.00 ± 0.01 | 11.00 ± 0.26 | 9.50 | 1.1584 | 1.3787 | 15.98 |
| N53 | 3.89 ± 0.02 | 2.00 ± 0.01 | 13.31 ± 0.21 | 10.97 | 1.2129 | 1.5045 | 19.38 |
| N54 | 3.55 ± 0.02 | 2.00 ± 0.01 | 11.95 ± 0.21 | 9.90 | 1.2066 | 1.4988 | 19.50 |
| N55 | 3.94 ± 0.02 | 2.00 ± 0.01 | 13.58 ± 0.32 | 11.13 | 1.2197 | 1.5014 | 18.76 |
| N56 | 3.79 ± 0.01 | 2.00 ± 0.01 | 12.84 ± 0.34 | 10.66 | 1.2049 | 1.4957 | 19.44 |
| N57 | 3.62 ± 0.02 | 2.00 ± 0.01 | 11.93 ± 0.32 | 10.12 | 1.1779 | 1.4121 | 16.58 |
| N58 | 3.35 ± 0.01 | 2.00 ± 0.01 | 10.74 ± 0.25 | 9.28 | 1.1580 | 1.4070 | 17.70 |
| N59 | 3.48 ± 0.01 | 2.00 ± 0.01 | 11.45 ± 0.43 | 9.68 | 1.1823 | 1.3887 | 14.86 |
| N60 | 3.14 ± 0.01 | 2.00 ± 0.01 | 10.06 ± 0.26 | 8.62 | 1.1674 | 1.3838 | 15.64 |
| N61 | 3.78 ± 0.01 | 2.00 ± 0.01 | 13.07 ± 0.38 | 10.63 | 1.2296 | 1.5106 | 18.60 |
| N62 | 3.13 ± 0.02 | 2.00 ± 0.01 | 10.26 ± 0.29 | 8.58 | 1.1950 | 1.5049 | 20.59 |
| N63 | 3.71 ± 0.01 | 2.00 ± 0.01 | 12.74 ± 0.42 | 10.41 | 1.2244 | 1.5075 | 18.78 |
| N64 | 3.11 ± 0.02 | 2.00 ± 0.01 | 10.25 ± 0.44 | 8.52 | 1.2028 | 1.5017 | 19.91 |

Table S6: µCT scan parameters for all the reconstructed and segmented formulations. Scan sequences are given as sequences of the format <number of scans>(acquisition rate of those scans). If the scan consists of multiple acquisition rates, they are listed in order, separated by a dash.

| **Formulation** | **CT data designation** | **Frames per scan** | **Total number of scans** | **Scan sequence** | **Delta (Paganin filter)** | **Beta (Paganin filter)** |
| --- | --- | --- | --- | --- | --- | --- |
| N1 | N1_1ms_500prj_WB50_w_01 | 500 | 120 | 120(0.2Hz) | 1.00E-07 | 5.00E-10 |
| N2 | N2_w_01 | 500 | 55 | 1-30(1Hz)-24(0.2Hz) | 1.00E-07 | 5.00E-10 |
| N3 | N3_w_01 | 500 | 55 | 1-30(1Hz)-24(0.2Hz) | 1.00E-07 | 5.00E-10 |
| N4 | N4_w_01 | 500 | 55 | 1-30(1Hz)-24(0.2Hz) | 1.00E-07 | 5.00E-10 |
| N5 | N5_w_02 | 500 | 31 | 1-30(1Hz)-24(0.2Hz) | 1.00E-07 | 5.00E-10 |
| N6 | N6_w_01 | 500 | 29 | 1-30(1Hz)-24(0.2Hz) | 1.00E-07 | 5.00E-10 |
| N7 | N7_w_03 | 500 | 8 | 8(0.02Hz) | 1.00E-07 | 5.00E-10 |
| N8 | N8_1ms_500prj_WB50_w_01 | 500 | 60 | 60(2Hz) continuous | 1.00E-07 | 5.00E-10 |
| N9 | N9_w_01 | 500 | 55 | 1-30(1Hz)-24(0.2Hz) | 1.00E-07 | 5.00E-10 |
| N10 | N10_w_01 | 500 | 55 | 1-30(1Hz)-24(0.2Hz) | 1.00E-07 | 5.00E-10 |
| N11 | N11_w_01 | 500 | 55 | 1-30(1Hz)-24(0.2Hz) | 1.00E-07 | 5.00E-10 |
| N12 | N12_w_01 | 500 | 55 | 1-30(1Hz)-24(0.2Hz) | 1.00E-07 | 5.00E-10 |
| N13 | N13_w_01 | 500 | 55 | 1-30(1Hz)-24(0.2Hz) | 1.00E-07 | 5.00E-10 |
| N14 | N14_w_01 | 500 | 55 | 1-30(1Hz)-24(0.2Hz) | 1.00E-07 | 5.00E-10 |
| N15 | N15_w_01 | 500 | 29 | 1-30(1Hz)-24(0.2Hz) | 1.00E-07 | 5.00E-10 |
| N16 | N16_w_01 | 500 | 15 | 1-30(1Hz)-24(0.2Hz) | 1.00E-07 | 5.00E-10 |
| N17 | N17_w_01 | 500 | 55 | 1-30(1Hz)-24(0.2Hz) | 1.00E-07 | 5.00E-10 |
| N18 | N18_w_01 | 500 | 55 | 1-30(1Hz)-24(0.2Hz) | 1.00E-07 | 5.00E-10 |
| N19 | N19_w_02 | 500 | 35 | 1-10(1Hz)-24(0.2Hz) | 1.00E-07 | 5.00E-10 |
| N20 | N20_w_01 | 500 | 35 | 1-10(1Hz)-24(0.2Hz) | 1.00E-07 | 5.00E-10 |
| N21 | N21_w_01 | 500 | 15 | 1-30(1Hz)-24(0.2Hz) | 1.00E-07 | 5.00E-10 |
| N22 | N22_w_01 | 500 | 10 | 1-30(1Hz)-24(0.2Hz) | 1.00E-07 | 5.00E-10 |
| N23 | N23_w_01 | 500 | 13 | 1-30(1Hz)-24(0.2Hz) | 1.00E-07 | 5.00E-10 |
| N24 | N24_w_01 | 500 | 11 | 1-30(1Hz)-24(0.2Hz) | 1.00E-07 | 5.00E-10 |
| N25 | N25_w_01 | 500 | 35 | 1-10(1Hz)-24(0.2Hz) | 1.00E-07 | 5.00E-10 |
| N26 | N26_w_01 | 500 | 35 | 1-10(1Hz)-24(0.2Hz) | 1.00E-07 | 5.00E-10 |
| N27 | N27_w_01 | 500 | 35 | 1-30(1Hz)-24(0.2Hz) | 1.00E-07 | 5.00E-10 |
| N28 | N28_w_02 | 500 | 55 | 1-30(1Hz)-24(0.2Hz) | 1.00E-07 | 5.00E-10 |
| N29 | N29_w_01 | 500 | 11 | 1-30(1Hz)-24(0.2Hz) | 1.00E-07 | 5.00E-10 |
| N30 | N30_w_01 | 500 | 8 | 1-30(1Hz)-24(0.2Hz) | 1.00E-07 | 5.00E-10 |
| N31 | N31_w_01 | 500 | 55 | 1-30(1Hz)-24(0.2Hz) | 1.00E-07 | 5.00E-10 |
| N32 | N32_w_01 | 500 | 10 | 1-30(1Hz)-24(0.2Hz) | 1.00E-07 | 5.00E-10 |
| N33 | N33_w_01 | 500 | 35 | 1-10(1Hz)-24(0.2Hz) | 1.00E-07 | 5.00E-10 |
| N34 | N34_w_01 | 500 | 35 | 1-10(1Hz)-24(0.2Hz) | 1.00E-07 | 5.00E-10 |
| N35 | N35_w_01 | 500 | 35 | 1-10(1Hz)-24(0.2Hz) | 1.00E-07 | 5.00E-10 |
| N36 | N36_w_01 | 500 | 35 | 1-10(1Hz)-24(0.2Hz) | 1.00E-07 | 5.00E-10 |
| N37 | N37_w_01 | 500 | 52 | 1-30(1Hz)-24(0.2Hz) | 1.00E-07 | 5.00E-10 |
| N38 | N38_w_01 | 500 | 33 | 1-30(1Hz)-24(0.2Hz) | 1.00E-07 | 5.00E-10 |
| N39 | N39_w_01 | 500 | 16 | 1-30(1Hz)-24(0.2Hz) | 1.00E-07 | 5.00E-10 |
| N40 | N40_w_01 | 500 | 24 | 1-30(1Hz)-24(0.2Hz) | 1.00E-07 | 5.00E-10 |
| N41 | N41_w_01 | 500 | 35 | 1-10(1Hz)-24(0.2Hz) | 1.00E-07 | 5.00E-10 |
| N42 | N42_w_01 | 500 | 35 | 1-10(1Hz)-24(0.2Hz) | 1.00E-07 | 5.00E-10 |
| N43 | N43_w_01 | 500 | 35 | 1-10(1Hz)-24(0.2Hz) | 1.00E-07 | 5.00E-10 |
| N44 | N44_w_01 | 500 | 35 | 1-10(1Hz)-24(0.2Hz) | 1.00E-07 | 5.00E-10 |
| N45 | N45_w_01 | 500 | 35 | 1-30(1Hz)-24(0.2Hz) | 1.00E-07 | 5.00E-10 |
| N46 | N46_w_01 | 500 | 22 | 1-30(1Hz)-24(0.2Hz) | 1.00E-07 | 5.00E-10 |
| N47 | N47_w_01 | 500 | 71 | 1-30(1Hz)-40(0.2Hz) | 1.00E-07 | 5.00E-10 |
| N48 | N48_w_02 | 500 | 55 | 1-30(1Hz)-24(0.2Hz) | 1.00E-07 | 5.00E-10 |
| N49 | N49_w_01 | 500 | 55 | 1-30(1Hz)-24(0.2Hz) | 1.00E-07 | 5.00E-10 |
| N50 | N50_w_01 | 500 | 55 | 1-30(1Hz)-24(0.2Hz) | 1.00E-07 | 5.00E-10 |
| N51 | N51_w_01 | 500 | 35 | 1-10(1Hz)-24(0.2Hz) | 1.00E-07 | 5.00E-10 |
| N52 | N52_w_01 | 500 | 35 | 1-10(1Hz)-24(0.2Hz) | 1.00E-07 | 5.00E-10 |
| N53 | N53_w_02 | 500 | 13 | 1-30(1Hz)-24(0.2Hz) | 1.00E-07 | 5.00E-10 |
| N54 | N54_w_01 | 500 | 13 | 1-30(1Hz)-24(0.2Hz) | 1.00E-07 | 5.00E-10 |
| N55 | N55_w_01 | 500 | 25 | 1-30(1Hz)-24(0.2Hz) | 1.00E-07 | 5.00E-10 |
| N56 | N56_w_01 | 500 | 22 | 1-30(1Hz)-24(0.2Hz) | 1.00E-07 | 5.00E-10 |
| N57 | N57_w_01 | 500 | 35 | 1-10(1Hz)-24(0.2Hz) | 1.00E-07 | 5.00E-10 |
| N58 | N58_w_01 | 500 | 35 | 1-10(1Hz)-24(0.2Hz) | 1.00E-07 | 5.00E-10 |
| N59 | N59_w_01 | 500 | 35 | 1-10(1Hz)-24(0.2Hz) | 1.00E-07 | 5.00E-10 |
| N60 | N60_w_01 | 500 | 35 | 1-10(1Hz)-24(0.2Hz) | 1.00E-07 | 5.00E-10 |
| N61 | N61_w_01 | 500 | 13 | 1-30(1Hz)-24(0.2Hz) | 1.00E-07 | 5.00E-10 |
| N62 | N62_w_01 | 500 | 11 | 1-30(1Hz)-24(0.2Hz) | 1.00E-07 | 5.00E-10 |
| N63 | N63_w_01 | 500 | 26 | 1-30(1Hz)-24(0.2Hz) | 1.00E-07 | 5.00E-10 |
| N64 | N64_w_01 | 500 | 16 | 1-30(1Hz)-24(0.2Hz) | 1.00E-07 | 5.00E-10 |

1. CNN training

The image stacks used in the training of the U-Net were generated from random tablet types at random time points, uniformly sampling the whole dataset. From each of these reconstructed CT images chosen at random, a subset of size 80x240x240 (z,y,x) was extracted from a random position within the z-axis. To generate the training data for set 1 (three segmentation categories, no inorganic tablet phase), a total of 96 such subsets were identified. The training data for set 2 (four segmentation categories, inorganic filler material) was created from 93 subsets.

The selected subsets of reconstructed CT images were segmented into a mask (class 4), background (class 3), organic tablet (class 1), and, where applicable, inorganic tablet (class 2) using Ilastik’s manually assisted pixel classification workflow. Due to the variance in image quality between tablet types and time points, each image stack had to be segmented individually.

Each image stack, along with its segmented counterpart, was subdivided into five continuous sets of 16 images to match the input dimensions of the convolutional neural network. That subdivision increased the total number of ground truth data stacks to 480 for set 1 and 465 for set 2. Finally, the reconstructed CT image stacks and their segmented counterparts were assigned to either the training or the validation set at a ratio of 3:1.

The U-Net created for the present segmentation task features 1’623’947 parameters, 1’622’443 of which are trainable. One input image stack has a total of 921’600 voxels. Due to GPU memory limitations, the training data sets had to be split into batches, each containing 40 stacks of 16x240x240 reconstructed images and their corresponding segmented ground truth data.

The networks were trained based on the progression of the validation loss metric. Specifically, the training was stopped and reverted to its best state if no improvement greater or equal to 0.001 was observed in the validation loss metric over 200 epochs.

The progression of loss and accuracy values during training of set 1 (3 segmentation classes, organic filler materials mannitol or cellulose) can be found in Figure S2. The progression of loss and accuracy values for the training of set 2 (4 segmentation classes, inorganic filler materials calcium carbonate or calcium phosphate) can be found in Figure S3.

The rate of improvement to training- and validation accuracy for set 1 slowed considerably after around epoch 200. Still, visual inspection of segmentation performance shows notable improvement past that point. Validation loss and accuracy values were very noisy compared to the training loss and accuracy values. Stopping criteria were reached for the training of set 1 after 1127 epochs. The state of the network was then reverted to its optimum in epoch 927, and the weights were saved to a file.

Similar observations could be made for the training of set 2. There was an additional notable improvement late in the training process, starting at epoch 400, after which the rate of improvement slowed down again. Similar to set 1, the validation loss and accuracy values were very noisy throughout the training. Stopping criteria for set 2 were reached after 1326 epochs, at which point its optimum state from epoch 1126 was restored and saved to file.

Figure S2: Training progression of the U-Net on the training- and validation data of set 1 (3 segmentation classes). The dotted blue line shows loss values and the solid green line validation loss values. The dotted red line shows accuracy values and the solid orange line shows validation accuracy values.

Figure S3: Training progression of the U-Net on the training- and validation data of set 2 (4 segmentation classes). The dotted blue line shows loss values, and the solid green line validation loss values. The dotted red line shows accuracy values, and the solid orange line shows validation accuracy values.

The training of the U-Net shows a relatively slow convergence rate and features noisy validation metrics, especially the validation loss values. The slow rate of training progression is not problematic since the irregular pattern of the validation loss function is likely indicative of the heterogeneous quality of the ground truth data coupled with comparatively small training batch sizes. The quality of the ground truth data varies from one image stack to the next, and the resulting trained network thus represents a compromise rather than a perfect solution. The noisy behavior of the performance metrics could be smoothed out by using more powerful computational hardware to run larger batch sizes or a distributed computing approach to neural network training. However, this was not deemed necessary, given the satisfactory segmentation performance of CNN.

A separate set of 120 manually segmented image stacks was created to independently measure network performance. To make this evaluation dataset, ten tablets and time points were identified at random for each U-Net. Each timepoint (200x240x240) was hand-segmented using Ilastik, as described previously. The z dimension was then split into 12 batches of size 16 to match the input dimensions of the neural network. Inference was run on each evaluation dataset, and the U-Net prediction was compared with the manual segmentation. A Confusion matrix was created for each U-Net (Table S7, S8), showing network performance as number of correctly assigned labels in the diagonal (where the U-Net predicted label matches the manually segmented ‘actual’ label) as well as the number of incorrectly identified labels (where the U-Net prediction differs from the ‘actual’ label based on manual segmentation) for each segmentation class across the whole evaluation dataset (all voxels from all image stacks). The confusion matrices confirm the heterogeneous quality of the image data, as noted during the visual inspection and analysis of training progression metrics. The assigned segmentation classes by the trained U-Nets match the ground truth in the majority of cases, but misclassifications still happen relatively often, especially between the background and the organic tablet component labels. This behavior was expected given the image quality of the reconstructed data and the fact that not even the machine learning-assisted image segmentation approach provided by Ilastik could provide one single ruleset that applies to the whole dataset. The output of the trained U-Nets holds up to visual inspection and numerical analysis of the results confirms that the segmentation quality is consistent enough for analyzing the disintegration process. This confirms the validity of our approach and highlights the need to use a deep learning approach for the present image segmentation task.

Table S7: Confusion matrix indicating the performance of the U-Net segmenting data from set 2 into 4 segmentation categories

|  |  | Predicted | | | |
| --- | --- | --- | --- | --- | --- |
|  |  | **Mask** | **Background** | **Organic** | **Inorganic** |
| Actual | **Mask** | 4.79E+07 | 2.91E+04 | 0 | 0 |
|  | **Background** | 7.33E+05 | 3.81E+07 | 1.96E+06 | 5.18E+05 |
|  | **Organic** | 139 | 1.04E+06 | 8.51E+06 | 5.75E+05 |
|  | **Inorganic** | 1 | 2.30E+05 | 3.71E+05 | 1.06E+07 |

Table S8: Confusion matrix indicating the performance of the U-Net segmenting data from set 2 into 4 segmentation categories

|  |  | Predicted | | |
| --- | --- | --- | --- | --- |
|  |  | **Mask** | **Background** | **Organic** |
| Actual | **Mask** | 4.67E+07 | 294 | 0 |
|  | **Background** | 1.64E+06 | 3.54E+07 | 2.22E+06 |
|  | **Organic** | 28 | 1.28E+06 | 2.33E+07 |

Table S9: DOE response parameters used in fitting the model describing formulations containing a swelling polymer. The intensity of erosion and swelling are subjective measures based on expert opinion, whereas the volumetric disintegration rate constant is an objective parameter automatically extracted from the image data. Positive values signify increase in tablet volume over time, negative value signify a decrease in tablet volume, normalized to the initial dry state tablet volume.

| **Formulation** | **Intensity of Erosion** [1 – 10] | **Intensity of Swelling** [1 - 10] | **Volumetric Swelling Rate Constant** [% of intact state] |
| --- | --- | --- | --- |
| N1 | 5 | 6.5 | 0.03002 |
| N2 | 3.5 | 5.5 | 0.00806 |
| N3 | 2.5 | 2 | 0.04727 |
| N4 | 3.5 | 3 | 0.04611 |
| N9 | 2 | 4 | 0.01403 |
| N10 | 3.5 | 4 | 0.06097 |
| N11 | 3 | 2.5 | 0.07951 |
| N1332 | 3 | 2.5 | 0.06966 |
| N17 | 2.5 | 6.25 | 0.04876 |
| N18 | 2.5 | 5.5 | 0.06409 |
| N19 | 3 | 3 | 0.02555 |
| N20 | 3.5 | 3 | 0.0671 |
| N25 | 2.5 | 7 | 0.02504 |
| N26 | 2 | 7.5 | 0.03422 |
| N27 | 3 | 4.5 | 0.17812 |
| N28 | 1.5 | 4.5 | 0.08347 |
| N33 | 2 | 3 | -0.05847 |
| N34 | 2.5 | 3.5 | 6.01E-04 |
| N35 | 3.5 | 2 | -0.02162 |
| N36 | 3.25 | 2 | 9.86E-04 |
| N41 | 2.5 | 4 | -0.07364 |
| N42 | 2.5 | 3.5 | -0.03664 |
| N43 | 2.5 | 2 | -0.00698 |
| N44 | 2.75 | 2 | 0.01038 |
| N49 | 4 | 6.5 | -0.1662 |
| N50 | 4 | 6.5 | -0.63271 |
| N51 | 4.25 | 1.5 | -0.10578 |
| N52 | 4 | 5 | -0.16288 |
| N57 | 4 | 7 | -0.17851 |
| N58 | 2.5 | 5.5 | -0.21872 |
| N59 | 4 | 2 | -0.09364 |
| N60 | 3.5 | 1.5 | -0.15449 |

Table S10: DOE response parameters used in fitting the model describing formulations containing a disintegrant. The intensity of erosion and swelling are subjective measures based on expert opinion, whereas the disintegration time is an objective parameter automatically extracted from the image data.

| **Formulation** | **Intensity of Erosion** [1 – 10] | **Intensity of Swelling** [1 - 10] | **Disintegration Time** [s] |
| --- | --- | --- | --- |
| N5 | 7 | 4.5 | 10.16544 |
| N6 | 8.5 | 2.5 | 10.33784 |
| N7 | 8.5 | 2.5 | 133.3333 |
| N8 | 6 | 1 | 241.42 |
| N13 | 4 | 3.5 | 900 |
| N14 | 7.75 | 3 | 13.73475 |
| N15 | 5.5 | 5 | 17.68562 |
| N16 | 6 | 5 | 11.15173 |
| N21 | 8.5 | 6 | 7 |
| N22 | 9 | 6 | 6.30389 |
| N23 | 6.75 | 7.5 | 9.09848 |
| N24 | 7.5 | 7 | 6.02692 |
| N29 | 8.25 | 6 | 6.70736 |
| N30 | 9.5 | 6.5 | 4.07061 |
| N31 | 7.25 | 6 | 11.16722 |
| N32 | 7.5 | 6 | 6.78003 |
| N37 | 4.5 | 3 | 133.3953 |
| N38 | 4.5 | 3.5 | 36.71831 |
| N39 | 4.25 | 6 | 13.80388 |
| N40 | 4.5 | 6 | 11.72493 |
| N45 | 6 | 1.5 | 49.99999 |
| N46 | 7 | 2 | 19.18521 |
| N47 | 4.5 | 2 | 900 |
| N48 | 2 | 2 | 900 |
| N53 | 7.5 | 3.5 | 8.20627 |
| N54 | 8 | 2 | 8.12067 |
| N55 | 6.5 | 4.75 | 20.12042 |
| N56 | 6 | 4.5 | 16.59964 |
| N61 | 7.5 | 1.5 | 8.16186 |
| N62 | 7.5 | 2.25 | 5.149 |
| N63 | 5 | 2 | 16.41585 |
| N64 | 5.5 | 2.5 | 12.44172 |


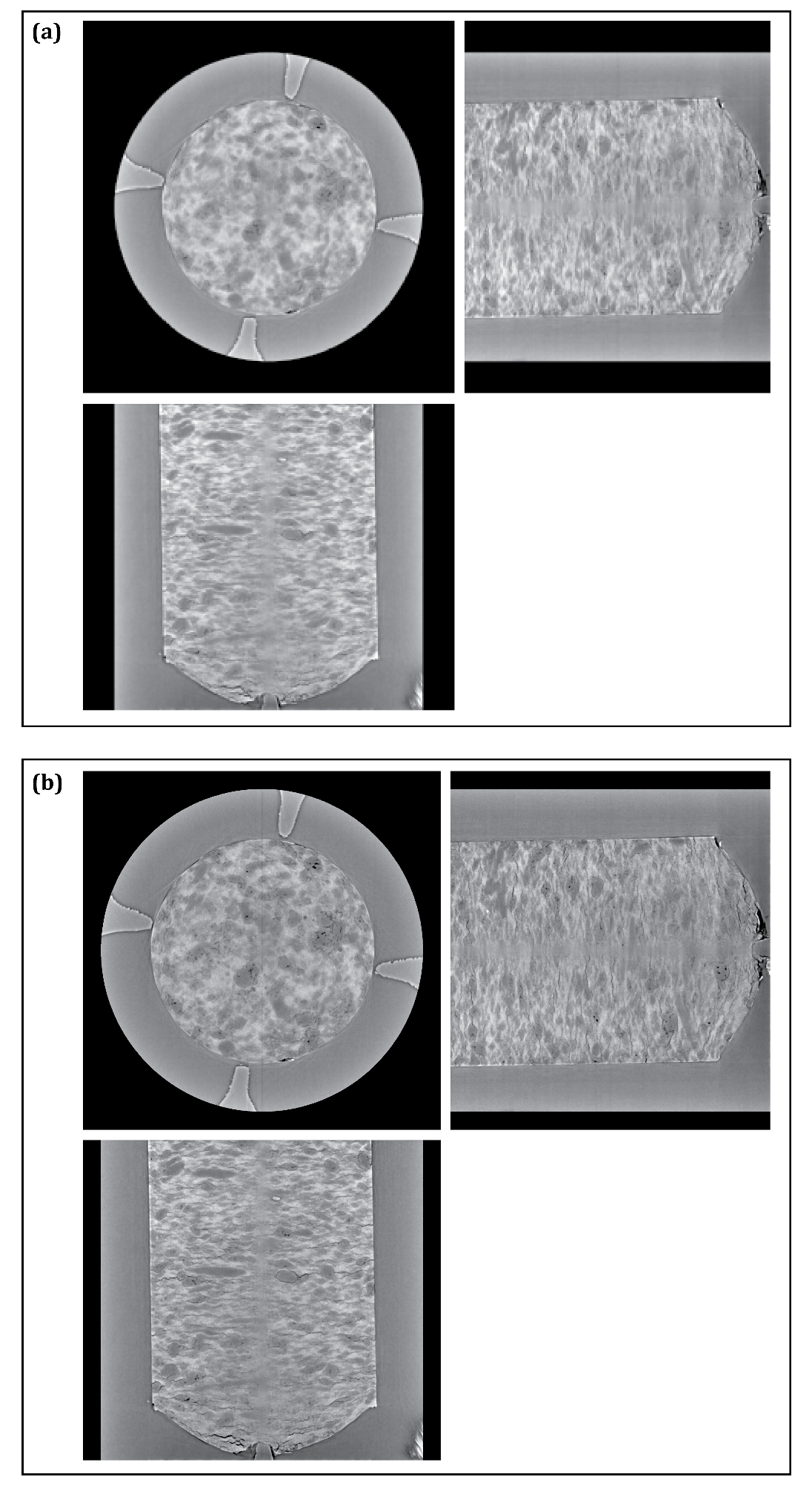


Figure S4: Orthogonal Views of the reconstructed image volume of formulation N2 at timepoint 0 after (a) and before (b) binning.


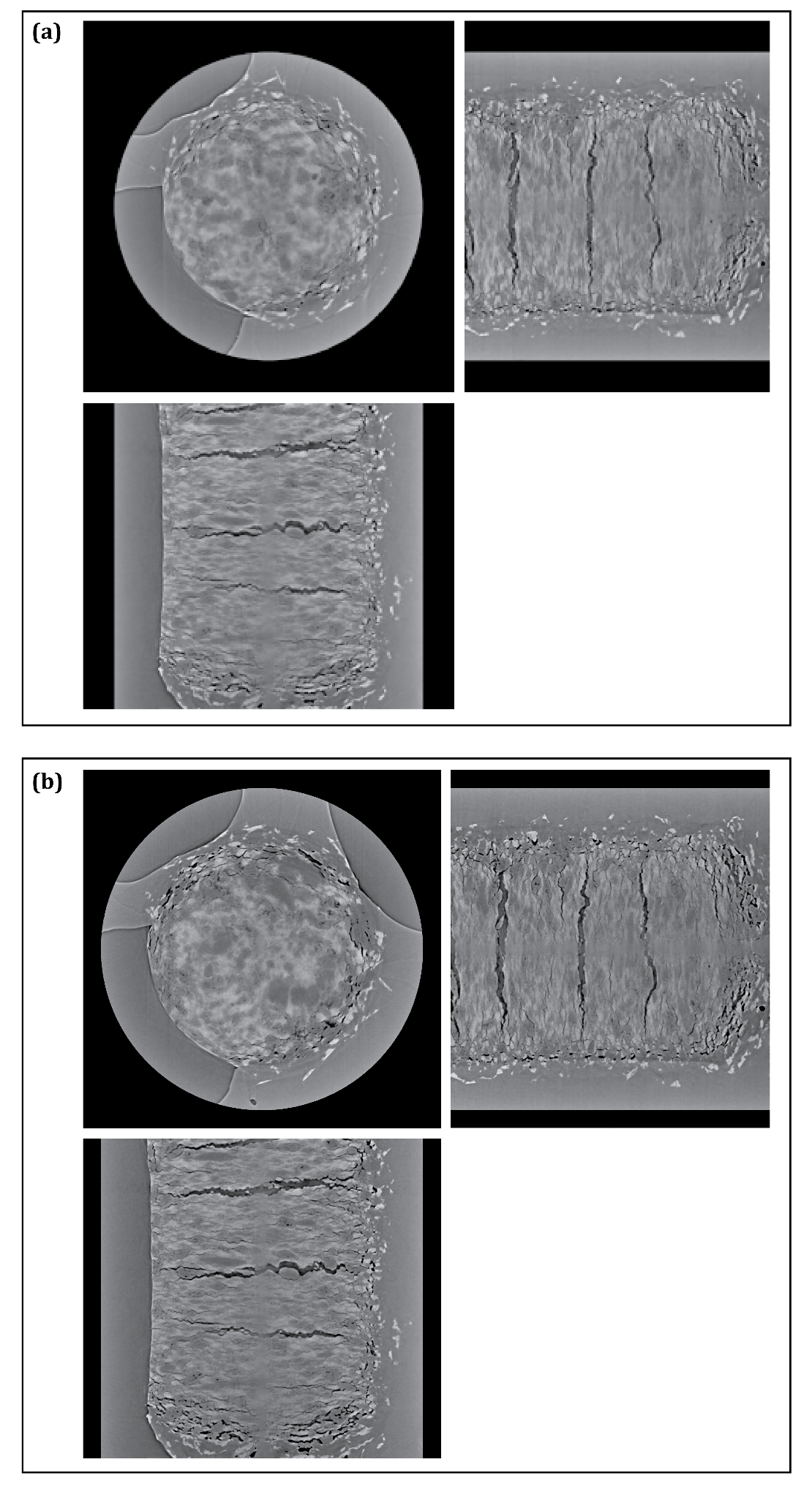


Figure S5: Orthogonal Views of the reconstructed image volume of formulation N2 at timepoint 0 after (a) and before (b) binning.
